# Distinct Phenotypes of Peripheral Innate Lymphoid Cells and T Cells in Type 2 and Non‐Type 2 Asthma

**DOI:** 10.1002/clt2.70108

**Published:** 2025-09-18

**Authors:** Maura M. Kere, Sophia Björkander, Simon Kebede Merid, Natalia Hernandez‐Pacheco, Paul Maier, Anne‐Sophie Merritt, Anna Bergström, Inger Kull, Carsten O. Daub, Jenny Mjösberg, Christopher Andrew Tibbitt, Erik Melén

**Affiliations:** ^1^ Department of Clinical Science and Education Södersjukhuset Karolinska Institutet Stockholm Sweden; ^2^ Department of Medicine Center for Infectious Medicine Karolinska Institutet Karolinska University Hospital Huddinge Stockholm Sweden; ^3^ Centre for Occupational and Environmental Medicine Stockholm Sweden; ^4^ Institute of Environmental Medicine Karolinska Institutet Stockholm Sweden; ^5^ Sachs' Children and Youth Hospital Södersjukhuset Stockholm Sweden; ^6^ Department of Biosciences and Nutrition Karolinska Institutet Stockholm Sweden; ^7^ Science for Life Laboratory Stockholm Sweden; ^8^ Clinical Lung‐ and Allergy Research Unit Medical Unit for Lung and Allergy Diseases Karolinska University Hospital Huddinge Stockholm Sweden

**Keywords:** asthma, CD4+ T cells, CD8+ T cells, innate lymphoid cells, non‐type 2, type 2, young adults

## Abstract

**Background:**

Investigation of T cell and innate lymphoid cell (ILC) subsets in type 2 (T2) and non‐type 2 (non‐T2) asthma are needed to elucidate disease mechanisms. In this study, we aimed to identify ILC, CD4+, and CD8+ T cell populations in blood that differentiate between T2 and non‐T2 features in subjects with and without asthma.

**Methods:**

The study population included 86 young adults selected from the Swedish population‐based BAMSE cohort. Asthma and non‐asthma subjects with sensitization to inhalant allergens and/or blood eosinophil count ≥ 0.3 × 10^9^/L were classified into T2 groups. Non‐T2 groups were defined by the absence of sensitization to inhalant allergens and blood eosinophil count < 0.3 × 10^9^/L. PBMC samples underwent 18‐parameter flow cytometry to identify ILC and CD4+ and CD8+ T cell populations. Logistic regression models were employed on normalized flow cytometry data after hierarchical clustering.

**Results:**

A higher frequency of CD4+ CRTH2+ T memory cells was associated with T2 features independent of asthma status. The frequency of CD62L+ ILC2s was higher and CD4+ KLRG1+ central memory T cells was lower specifically in T2 asthma. Non‐T2 asthma was associated with increased frequencies of CD45RO+ ILC2s and CD8+ memory T cells.

**Conclusion:**

Our results suggest that T2 asthma and non‐T2 asthma are characterized by distinct features related to ILC and T cell populations. Further investigation of particularly ILC and CD8+ T cell subsets in non‐T2 asthma could offer a deeper understanding of underlying disease mechanisms for this endotype.

**Abbreviations**
BAMSE: Children (Barn), Allergy, Milieu, Stockholm, Epidemiology
BF%: body fat percentage
BMI: body mass index
CCR4: C‐C motif chemokine receptor 4
CRTH2: chemoattractant receptor‐homologous molecule
ERS/ATS: European Respiratory Society/American Thoracic Society
FeNO: fractional exhaled nitric oxide
FEV1: forced expiratory volume in 1 s
FVC: forced vital capacity
GINA: Global Initiative for Asthma
GLI: Global Lung Initiative
IgE: Immunoglobulin E
IL‐4RA: IL‐4 receptor
IL‐x: Interleukin x
ILC: innate lymphoid cell
NK: Natural Killer
OR: odds ratio
RV: representative variable
ST2: Suppression of Tumorigenicity 2 protein
T2: Type 2
TCM: T central memory cells
TEM: T effector memory cells
Tc2: CD8+ type 2 cytotoxic T cells
Thx: CD4+ type x T helper cells

## 1 Introduction

Type 2 (T2) immune responses contribute to our defense against pathogens, but when dysregulated, can trigger the development of diseases in several organ systems [1]. T2 diseases share functional mechanisms (endotype) and include, for example, asthma, atopic dermatitis, allergic rhinitis and conjunctivitis, as well as chronic rhinosinusitis [1]. Asthma can be divided into T2 and non‐T2 endotypes based on the underlying inflammatory processes in the airways. Typically, IgE‐sensitization, fractional exhaled nitric oxide (FeNO), and blood or sputum eosinophils are used to classify asthma patients into these endotypes.

Several pathways and immune cells drive T2 asthma and typically involve T2 T helper (Th2) cells and T2 innate lymphoid cells (ILC2s) as central mediators of inflammation, giving rise to eosinophilia and IgE‐sensitization [2, 3]. The release of T2 cytokines (IL‐4, IL‐5, IL‐9, and IL‐13) from T2 cells in response to alarmin release from damaged airway epithelium and allergen challenge leads to the typical T2 manifestations [2, 4]. ILC2s are antigen‐independent immune cells regarded as innate counterparts of Th2 cells, potent producers of T2 cytokines and thus, drivers of T2 inflammation [4, 5, 6]. The dysregulation of ILC2 in the airways can lead to harmful effects, such as initiation of airway inflammation and subsequent asthma development, or incitement of asthma exacerbations [5, 7]. ILC2s are reportedly elevated in absolute and relative numbers and more active through the production of increased levels of T2 cytokines in peripheral blood, sputum, and bronchoalveolar lavage fluid in individuals with asthma [6].

The role of CD8+ T cells in asthma has been under debate [8]. However, cytotoxic CD8+ T (Tc) cells can respond to typical T2 signals and produce T2 cytokines (referred to as Tc2 cells) in asthma [9]. Elevated CD8+ T cell levels in bronchial biopsies have been associated with lung function decline over time in individuals with asthma [10]. CD8+ T cells may exhibit T2 features such as expression of chemoattractant receptor‐homologous molecule type 2 (CRTH2; cellular marker for T2 cells) and release of IL‐4, ‐5, and ‐13 [11].

Although Th2 cells and to an extent ILC2s have an established role in T2 asthma, the role of Tc2 cells is less clear [11]. Further, the involvement of other T‐ and ILC subsets in non‐T2 asthma has not been extensively investigated. Through our inclusion of subjects both with and without T2 features among the non‐asthmatic controls, we have the opportunity to disentangle cellular characteristics related to T2 features specific to asthma. Thus, we aimed to identify differential ILC, CD4+, and CD8+ T cell populations in blood between T2 and non‐T2 features in subjects with and without asthma, to elucidate mechanisms and ultimately improve diagnostics and treatment of common asthma endotypes.

## 2 Methods

### 2.1 Study Population

The study population consisted of 86 young adults participating in the ongoing, population‐based Swedish BAMSE (abbreviated from Children/Barn (barn = Swedish for children), Allergy, Milieu, Stockholm, Epidemiology) birth cohort [12, 13]. Included subjects were participants of the BAMSE COVID‐19 follow‐up Phase 2, originally selected as previously described [14], and for this study classified as detailed in Supporting Information S1: Figure S1 [14]. For this study, we used questionnaire data from the BAMSE 24‐year follow‐up (2016‐2019) [15], and the first two phases of the BAMSE COVID‐19 follow‐up (BAMSE 26‐year follow‐up, 2020‐2021) [12, 16]. Data from clinical examinations was derived from the 24‐year follow‐up or Phase 2 of the COVID‐19 follow‐up. Informed written consent was obtained from all subjects, and ethical permits were granted by the Regional Ethics Review Board in Stockholm (nr: 2020‐02922 and 2024‐06052‐02).

### 2.2 Definitions of Subgroups

Subjects with asthma (*n* = 40) had a self‐reported doctor's diagnosis ever of asthma with concomitant breathing difficulties and/or asthma medication use in the last 12 months reported in the questionnaires collected at Phase 1 or Phase 2 of the BAMSE COVID‐19 follow‐up, consistent with asthma definitions used in several previous birth cohort studies [13, 14, 17, 18]. T2 asthma was defined as the combination of asthma with IgE‐sensitization to inhalant allergens (positive if inhalant‐allergen‐specific IgE‐value in serum was ≥ 0.35 kU/L) and/or blood eosinophil count ≥ 0.3 × 10^9^/L (eosinophil cut‐off suggested by the European Respiratory Society [19]; see Supporting Information S1: Table S1). Non‐T2 asthma was defined as asthma without IgE‐sensitization to inhalant allergens and blood eosinophil count < 0.3 × 10^9^/L. Identical definitions were used for subjects without asthma (*n* = 46), referred to as controls with or without T2 features, called T2 and non‐T2 controls.

### 2.3 Variable Description

During the 24‐year follow‐up, information on atopic eczema and rhinitis was collected by a questionnaire, and specific IgE levels were measured in serum collected during the clinical examination. Sensitization for food and inhalant allergen‐specific IgE was analyzed using the ImmunoCAP System (Thermo Fisher Scientific, Uppsala, Sweden) [20].

During Phase 2 of the BAMSE COVID‐19 follow‐up, data on current smoking and asthma severity proxies, such as activity limitation, nocturnal awakenings, and use of oral steroids due to breathing difficulties, were collected through a questionnaire (Supporting Information S1: Table S2). During the clinical examination of Phase 2, BMI and body fat percentage (BF%) were measured using a bioelectrical impedance machine (Tanita MC 780 body composition monitor) and lung function measurements, namely forced expiratory volume in 1 s, forced vital capacity, and their ratio (FEV1, FVC, and FEV1/FVC, respectively) were assessed through spirometry according to European Respiratory Society/American Thoracic Society (ERS/ATS) criteria using the Jaeger spirometry apparatus and SentrySuite 2.17 [21]. The measurements were transformed to z‐scores based on references from the Global Lung Initiative (GLI) [22]. Finally, blood cell counts were determined by routine flow cytometry at the Karolinska University Laboratory.

### 2.4 Flow Cytometry

We used 18‐parameter flow cytometry to analyze ILC, CD4+, and CD8+ T cell subpopulations in peripheral blood mononuclear cell (PBMC) samples obtained during the clinical examination of Phase 2 of the COVID‐19 follow‐up [14]. Blood from venipuncture was collected in sodium heparin tubes (BD Vacutainer). PBMCs were isolated by FICOLL density centrifugation using SepMate‐tubes (STEMCELL Technologies), diluted in 90% FBS + 10% dimethyl sulfoxide (DMSO), and stored at −150°C. PBMCs were thawed at 37°C and washed with pre‐warmed media. Dead Cell Marker (DCM) and a cocktail of surface antibodies were used to stain cells for 30 min at 4°C. Cells were fixed with 2% paraformaldehyde before acquisition with an LSRFortessa (BD Bioscience), and data was analyzed using Flowjo version 10.8.1 (BD) [14].

### 2.5 Statistical Methods

All statistical analyses were performed using R version 4.3.3 [23]. Clinical characteristics were investigated by using group‐wise statistics from the tableone R package. All discrete variables were analyzed by Fisher's exact test, and continuous, non‐normally distributed variables were analyzed by Mann‐Whitney *U* test (two groups) or Kruskal‐Wallis test (four groups). Normally distributed continuous variables were analyzed by *t*‐test (two groups) or ANOVA (four groups).

**FIGURE 1:**
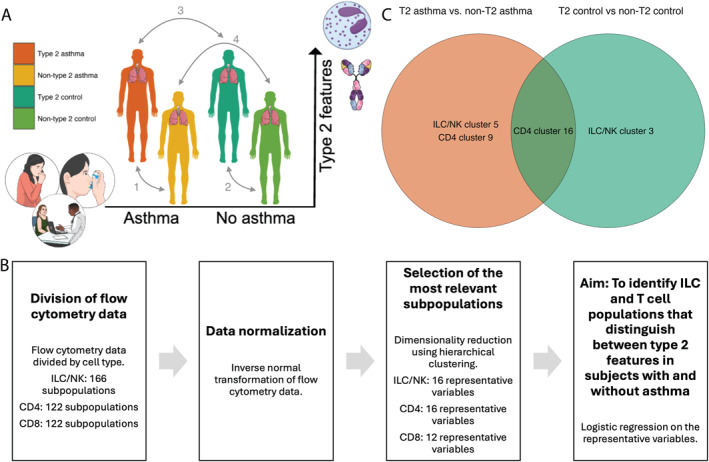
Study design (A), flow chart illustrating the workflow (B), and overlap of clusters that significantly differentiate type 2 (T2) and non‐T2 asthma or T2 and non‐T2 controls (C). (A) T2 asthma (*n* = 30) was defined as asthma in combination with IgE‐sensitization to inhalant allergens and/or blood eosinophil count ≥ 0.3 × 10^9^/L. Non‐T2 asthma (*n* = 10) was defined as asthma without IgE‐sensitization to inhalant allergens and blood eosinophil count < 0.3 × 10^9^/L. Identical definitions were used for subjects without asthma (*n* = 46), referred to as T2 controls (*n* = 20) and non‐T2 controls (*n* = 26). Comparison set 1 was employed to identify lymphocytes that distinguish between T2 and non‐T2 asthma. Comparison set 2 was employed to identify lymphocytes associated with T2 features in individuals without asthma. Comparison set 3 was employed to identify lymphocytes associated with T2 features in asthma but not in corresponding controls. Comparison set 4 was employed to identify lymphocytes associated with non‐T2 asthma. (B) Flow cytometry data was divided by cell type into ILC/NK cells (166 subpopulations), CD4 T cells (122 subpopulations), and CD8 T cells (122 subpopulations) and flow cytometry data was inverse normally transformed. The most relevant subpopulations within each cell type were selected by hierarchical clustering, employing the Ward D2 method on a Euclidean distance matrix for dimensionality reduction. Hierarchical clustering produced 16 ILC/NK cell clusters, 16 CD4 T cell clusters, and 12 CD8 T cell clusters, and the cluster average was subsequentially used as a representative variable (RV) for each cluster. To identify lymphocyte subpopulations that differentiate between type 2 and non‐type 2 features in subjects with and without asthma, we employed logistic regression models, separately for each cell type, adjusted for sex, BMI (lean or overweight/obese), smoking (self‐reported; yes/no), and blood lymphocyte count. (C) Overlap of clusters that significantly differentiate type 2 (T2) and non‐T2 asthma or T2 and non‐T2 controls (from the logistic regression models between comparison sets 1 and 2, as described above).

In the exploratory analyses, flow cytometry data were divided by cell type into ILC/NK cells (166 subpopulations), CD4 T cells (122 subpopulations), and CD8 T cells (122 subpopulations) [14], (the analytical workflow is illustrated in Figure 1B). The flow cytometry data was inverse‐normally transformed to ensure normality and similar scales between different parameters. Secondly, the most relevant subpopulations within each cell type were selected by hierarchical clustering, employing the “Ward.D2” method on an Euclidean distance matrix to generate dendrograms and perform dimensionality reduction. The resulting dendrograms were then cut at a height of 20 which was determined by visual inspection (Supporting Information S1: Figure S2). Hierarchical clustering produced 16 ILC/NK cell clusters, 16 CD4 T cell clusters, and 12 CD8 T cell clusters, and the cluster average was subsequently used as a representative variable (RV) for each cluster (see Supporting Information S1: Tables S4 and S9–S11).

To identify lymphocyte subpopulations that differentiate between T2 and non‐T2 features in subjects with and without asthma, we employed logistic regression models separately for each cell type, adjusted for sex, BMI (lean or overweight/obese according to definitions from the World Health Organization [24, 25]), smoking (self‐reported; yes/no), and blood lymphocyte count (×10^9^/L). First, we aimed to identify lymphocytes that are associated with T2 asthma when compared to non‐T2 asthma (comparison set 1 in Figure 1A). Second, we aimed to identify lymphocytes associated with T2 features in non‐asthmatics (T2 control vs. non‐T2 control; comparison set 2 in Figure 1A). Third, we aimed to identify lymphocytes specific to T2 features in asthma but not in controls (T2 asthma vs. T2 control; comparison set 3 in Figure 1A). Finally, we aimed to identify lymphocytes associated with non‐T2 asthma (non‐T2 asthma vs. non‐T2 control; comparison set 4 in Figure 1A). A *p*‐value of less than 0.05 was interpreted as significant.

## 3 Results

### 3.1 Allergic Comorbidities Differ Between T2 and Non‐T2 Groups With and Without Asthma

Of a total of 40 subjects with asthma, 30 (75%) subjects had T2 asthma and 10 (25%) had non‐T2 asthma according to our definition (Table 1). Among 46 subjects without asthma, 20 (43%) were defined as T2 controls and 26 (57%) as non‐T2 controls (Table 1). The most prevalent T2 feature among the T2 groups was sensitization to inhalant allergens only (Supporting Information S1: Table S1).

**TABLE 1:** Clinical characteristics of asthma and control groups with type 2 and non‐type 2 features.

	Asthma (*n* = 40)			Controls (*n* = 46)			*p*‐value (all)
	Type 2 asthma (*n* = 30)	Non‐type 2 asthma (*n* = 10)	*p*‐value	Type 2 control (*n* = 20)	Non‐type 2 control (*n* = 26)	*p*‐value	
Female sex (yes)	20 (66.7)	8 (80.0)	0.693^a^	14 (70.0)	18 (69.2)	1.000^a^	0.914^a^
Age	25.84 [25.09, 26.30]	25.68 [25.12, 25.99]	0.803^b^	25.67 [24.73, 26.44]	25.71 [25.31, 26.55]	0.451^b^	0.859^c^
BMI	24.35 [21.05, 27.58]	24.00 [22.35, 27.60]	0.791^b^	26.15 [21.55, 29.28]	24.85 [22.60, 28.08]	0.956^b^	0.793^c^
Overweight/obese^g^ (yes)	15 (50.0)	4 (40.0)	0.721^a^	13 (65.0)	12 (46.2)	0.244^a^	0.528^a^
Body fat percentage	24.60 [22.33, 34.60]	25.75 [22.37, 32.45]	0.628^b^	27.55 [23.88, 34.48]	26.60 [24.20, 34.77]	0.782^b^	0.707^c^
Current smoker (yes)	6 (20.0)	2 (20.0)	1.000^a^	4 (20.0)	2 (7.7)	0.380^a^	0.524^a^
Eczema^f^ (yes)	16 (53.3)	1 (10.0)	**0.026** ^a^	3 (15.0)	4 (15.4)	1.000^a^	**0.003** ^a^
Rhinitis^f^ (yes)	23 (76.7)	5/9 (55.6)	0.238^a^	9 (45.0)	3/25 (12.0)	**0.019** ^a^	**< 0.001** ^a^
IgE‐sensitization to food allergen^f^ (yes)	9 (30.0)	0 (0.0)	0.081^a^	2 (10.0)	0 (0.0)	0.184^a^	**0.003** ^a^
IgE‐sensitization to inhalant allergen^f^ (yes)	28 (93.3)	0 (0.0)	**< 0.001** ^a^	13 (65.0)	0 (0.0)	**< 0.001** ^a^	**< 0.001** ^a^
FEV1 *z*‐score	−0.11 (0.98)	−0.98 (1.38)	**0.036** ^d^	−0.09 (1.06)	−0.13 (0.93)	0.881^d^	0.114^e^
FVC *z*‐score	0.22 (0.92)	−0.39 (0.88)	0.076^d^	0.12 (0.94)	0.15 (0.79)	0.905^d^	0.303^e^
FEV1/FVC *z*‐score	−0.52 (1.04)	−0.86 (1.54)	0.430^d^	−0.37 (0.99)	−0.51 (0.75)	0.575^d^	0.666^e^
Blood eosinophils ≥ 0.3 × 10^9^/L (yes)	10 (33.3)	0 (0.0)	**0.043** ^a^	8 (40.0)	0 (0.00)	**< 0.001** ^a^	**< 0.001** ^a^
Blood cell count (×10^9^/L)							
Neutrophils	3.65 [2.82, 4.53]	2.70 [2.08, 3.78]	0.091^b^	4.00 [2.95, 5.35]	3.40 [3.02, 4.00]	0.287^b^	0.194^c^
Leukocytes	6.55 [5.62, 7.30]	5.25 [4.85, 6.05]	**0.041** ^b^	6.85 [5.95, 8.30]	6.10 [5.53, 6.97]	0.064^b^	**0.030** ^c^
Lymphocytes	2.05 [1.63, 2.40]	1.95 [1.80, 2.08]	0.510^b^	2.15 [1.87, 2.50]	2.05 [1.70, 2.58]	0.885^b^	0.588^c^
Monocytes	0.50 [0.40, 0.60]	0.45 [0.32, 0.50]	0.061^b^	0.50 [0.48, 0.65]	0.50 [0.40, 0.60]	0.298^b^	0.115^c^

*Note:* Discrete variables, indicated by “(yes)”, are presented as *n* (%); if the answer frequency was < 100% for discrete variables, the total number of subjects that replied is indicated after/. Non‐normally distributed continuous variables are presented as median [interquartile range] and normally distributed continuous variables are presented as mean (standard deviation). Nominally significant results are bolded (*p*‐value < 0.05).

Abbreviations: BMI, body mass index; FEV1, forced expiratory volume in 1 s; FVC, forced vital capacity; IgE, immunoglobulin E.

^a^ Fisher's exact test.

^b^ Mann‐Whitney *U* test.

^c^ Kruskal‐Wallis rank‐sum test.

^d^ *t*‐test.

^e^ ANOVA.

^f^ evaluated at the BAMSE 24‐year follow‐up.

^g^ BMI ≥ 25.0.

Comparing all four groups, we observed the highest prevalences of eczema, rhinitis, and IgE‐sensitization to food allergens in the T2 asthma group, whereas T2 controls had a significantly higher prevalence of rhinitis compared to non‐T2 controls (Table 1). Subjects with non‐T2 asthma had lower FEV1 *z*‐scores compared to subjects with T2 asthma, although other asthma severity proxies, such as inhaled or oral steroid use, did not differ between the asthma groups (Supporting Information S1: Table S2). The highest prevalence of persistent childhood asthma (asthma at 1, 2 or 4 years of age, and at 8, 12 or 16 years of age) was observed in the non‐T2 asthma group, whereas the highest prevalence of rhinitis and IgE‐sensitization to inhalant allergens over time was seen for the T2 asthma group, followed by the T2 control group (Supporting Information S1: Table S3). Although some control subjects had a history of childhood asthma, they were classified as controls at inclusion due to the absence of respiratory symptoms and medication use in the prior year.

### 3.2 CD62L+ ILC2s and KLRG1+ Memory T Cells Associate Uniquely With T2 Asthma, While Increased CRTH2+ Memory T Cells Associate With T2 Features

First, we aimed to identify lymphocytes associated with T2 features in subjects with and without asthma (T2 asthma compared to non‐T2 asthma, and T2 controls compared to non‐T2 controls; comparison sets 1 and 2 in Figure 1A). From these regression models, the representative variables (RVs, i.e., the cluster average for each cluster after hierarchical clustering) for CD4 cluster 16 (6.63 [1.56–28.20]) (OR [95% confidence interval]) and ILC/NK cluster 5 (4.24 [1.21–14.82]) were significantly positively associated with T2 asthma, whereas CD4 cluster 9 RV (0.28 [0.09–0.89]) was negatively associated with T2 asthma (Table 2; Supporting Information S1: Table S5). Similarly, the RVs for CD4 cluster 16 (2.19 [1.06–4.50]) and ILC/NK cluster 3 (4.86 [1.36–17.39]) were significantly positively associated with T2 controls (Table 2; Supporting Information S1: Table S6). Of these, only CD4 cluster 16 was associated with T2 features in both asthma and control groups (Figure 1C).

**TABLE 2:** Nominally significant results from four logistic regression models with representative variables (RVs) as predictors.

Cluster	Odds ratio [95% confidence interval]	*p*‐value
Comparison set 1: T2 asthma versus non‐T2 asthma		
ILC/NK Cluster 5 RV	4.24 [1.21–14.82]	0.024
CD4 Cluster 16 RV	6.63 [1.56–28.20]	0.010
CD4 Cluster 9 RV	0.28 [0.09–0.89]	0.031
Comparison set 2: T2 control versus non‐T2 control		
ILC/NK Cluster 3 RV	4.86 [1.36–17.39]	0.015
CD4 Cluster 16 RV	2.19 [1.06–4.50]	0.033
Comparison set 3: T2 asthma versus T2 control		
CD8 Cluster 6 RV	0.35 [0.13–0.97]	0.043
Comparison set 4: Non‐T2 asthma versus non‐T2 control		
ILC/NK Cluster 7 RV	5.71 [1.15–28.34]	0.033
CD4 Cluster 14 RV	3.06 [1.02–9.22]	0.046
CD8 Cluster 2 RV	3.71 [1.06–13.04]	0.041

*Note:* All models were adjusted for sex, BMI (lean vs. overweight/obese), smoking (yes/no), and blood lymphocyte count (×10^9^/L). Full results are shown in Supporting Information S1: Tables S4–S7.

Abbreviation: RV, representative variable.

**FIGURE 2:**
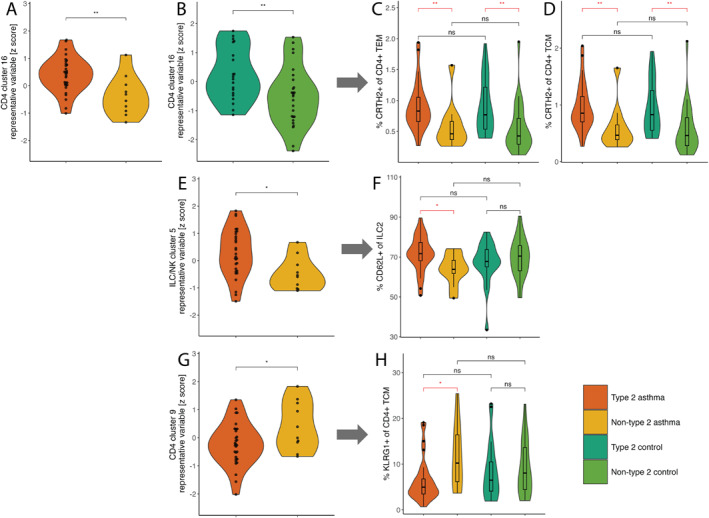
Selected significant associations from logistic regression models presented in Table 2 (A, B, E, G: Note that the *y*‐axis is inverse normally transformed) and selected individual subpopulations from each cluster that significantly differentiate between the groups (C, D, F, H: *p*‐values determined by Mann‐Whitney *U* test). (A–B) CD4 cluster 16 representative variable (RV) when contrasting T2 asthma and non‐T2 asthma (comparison set 1) (A) or T2 controls and non‐T2 controls (B). (C–D) The frequencies of CRTH2+ cells among CD4+ T effector (TEM) and central memory (TCM) cells (C and D, respectively). All subpopulations in this cluster are shown in Supporting Information S1: Figure S3. (E) ILC/NK cluster 5 RV when contrasting T2 asthma and non‐T2 asthma (comparison set 1). (F) The frequency of CD62L+ cells among type 2 innate lymphoid cells (ILC2s). All subpopulations in this cluster are shown in Supporting Information S1: Figure S4. (G) CD4 cluster 9 RV when contrasting T2 asthma and non‐T2 asthma (comparison set 1). (H) The frequency of KLRG1+ cells among CD4+ T central memory (TCM) cells. All subpopulations in this cluster are shown in Supporting Information S1: Figure S5. Ns: non‐significant. *p*‐value (*p* > 0.05); *: *p* ≤ 0.05, **: *p* ≤ 0.01.

We then examined the individual subpopulations in these clusters; in CD4 cluster 16, we identified a significantly higher frequency of CD4+ CRTH2+ cells among T effector memory (TEM) and T central memory (TCM) cells in T2 groups with and without asthma (Figure 2A–D; Supporting Information S1: Figure S3). In ILC/NK cluster 5, we noted a higher frequency of CD62L+ cells among ILC2s in T2 asthma compared to non‐T2 asthma (Figure 2E,F; Supporting Information S1: Figure S4). In contrast, in CD4 cluster 9, we observed a lower frequency of CD4+ KLRG1+ cells among TCM cells in T2 asthma compared to non‐T2 asthma (Figure 2G,H; Supporting Information S1: Figure S5). Finally, we examined the individual subpopulations in ILC/NK cluster 3 and detected a higher frequency of CD117‐ CD62L+ cells among ILCs in T2 controls compared to non‐T2 controls (Supporting Information S1: Figures S6A and S7). In summary, a higher frequency of CD4+ CRTH2+ TEM and TCM Th2 cells was associated with T2 features independent of asthma status, representing a common T2‐feature‐associated signal. However, the frequency of CD62L+ ILC2s was higher, and CD4+ KLRG1+ cells among TCM cells was lower only in T2 subjects with asthma.

Additionally, we aimed to identify lymphocytes specific to T2 features in asthma but not in controls by comparing T2 asthma to T2 controls (comparison set 3 in Figure 1A), and the RV for CD8 cluster 6 was negatively associated (0.35 [0.13–0.97]) with T2 asthma (Table 2; Supporting Information S1: Table S7) when compared to T2 controls. Exploring the individual subpopulations in this cluster, we did not observe any significant differences between the groups (Supporting Information S1: Figures S6B and S8).

### 3.3 Increased CD8+ CD45RO+ Memory T Cells and CD45RO+ ILC2s Characterize Non‐T2 Asthma

Then, we sought to identify lymphocytes that are associated specifically with non‐T2 asthma (non‐T2 asthma compared to non‐T2 controls; comparison set 4 in Figure 1A). From this regression model, the representative variables (RVs) for CD8 cluster 2 (3.71 [1.06–13.04]), ILC/NK cluster 7 (5.71 [1.15–28.34]), and CD4 cluster 14 (3.06 [1.02–9.22]) were positively associated with non‐T2 asthma (Table 2; Supporting Information S1: Table S8) when compared to non‐T2 controls.

**FIGURE 3:**
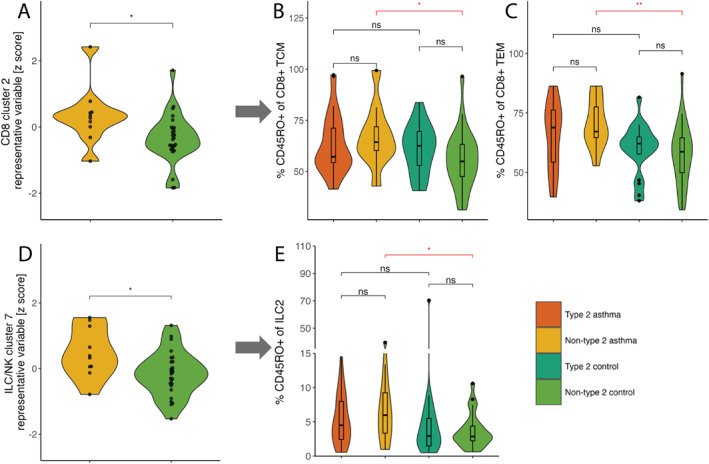
Selected significant associations from logistic regression models presented in Table 2 (A and D: note that the *y*‐axis is inverse normally transformed) and selected individual subpopulations from each cluster that significantly differentiate between the groups (B, C, and E: *p*‐values determined by Mann‐Whitney *U* test). (A) CD8 cluster 2 representative variable (RV) when contrasting non‐T2 asthma and non‐T2 controls (comparison set 4). (B–C) The frequencies of CD45RO+ cells among CD8+ T effector (TEM) and central memory (TCM) cells (B and C, respectively). All subpopulations in this cluster are shown in Supporting Information S1: Figure S9. (D) ILC/NK cluster 7 representative variable (RV) when contrasting non‐T2 asthma and non‐T2 controls (comparison set 4). (E) The frequency of CD4RO + cells among type 2 innate lymphoid cells (ILC2S). All subpopulations in this cluster are shown in Supporting Information S1: Figure S10. Ns: non‐significant *p*‐value (*p* > 0.05); *p ≤ 0.05, **p ≤ 0.01.

We explored the individual subpopulations in CD8 cluster 2 and ILC/NK cluster 7 and observed that the frequency of CD8+ CD45RO+ cells among TCM and TEM cells as well as CD45RO+ cells among ILC2s, was higher in non‐T2 asthma compared to non‐T2 controls (Figure 3A–E; Supporting Information S1: Figures S9 and S10). For CD4 cluster 14 we did not observe any significant differences in the individual subpopulations (Supporting Information S1: Figures S6C and S11). To summarize, a higher frequency of CD8+ CD45RO+ cells among TCM and TEM cells and CD45RO+ cells among ILC2s was observed uniquely in non‐T2 asthma.

## 4 Discussion

Herein, we sought to identify lymphocytes associated with T2 features in subjects with and without asthma and to identify lymphocytes associated specifically with non‐T2 asthma in young Swedish adults from the population‐based BAMSE cohort. We report a higher frequency of CD62L+ ILC2s and a lower frequency of CD4+ KLRG1+ cells among TCM cells specifically in T2 asthma. Also, we observed an association with an increased frequency of CD4+ CRTH2+ memory T cells with T2 features independent of asthma status, representing a common T2‐feature‐associated signal. Importantly, our study particularly highlights the changes in phenotypes observed in ILCs in individuals with the non‐T2 asthma endotype: a higher frequency of CD45RO+ cells among ILC2s and CD8+ memory T cells distinguished non‐T2 asthma from non‐T2 controls. These subsets have not been extensively investigated in the context of asthma, especially in individuals presenting with a non‐T2 endotype, and warrant further investigation, as fewer mechanisms behind non‐T2 asthma are known.

There is a paucity of studies that have performed deep immune phenotyping of non‐T2 asthma [26]. Interestingly, we found unique cellular associations in non‐T2 asthma that included higher CD45RO+ cells among ILC2s and CD8+ memory T cells when compared to non‐T2 controls. CD45 is a receptor tyrosine phosphatase found on T cells and ILCs. Naïve ILC and T cells express CD45RA, but after a challenge by an extrinsic stimuli and subsequent activation cells switch to CD45RO expression, which serves as a marker of memory status [27, 28]. To our knowledge, the association of a higher frequency of CD45RO+ cells among ILC2s in non‐T2 asthma has not been reported elsewhere to date. The increased frequency of CD45RO+ ILC2 within the non‐T2 asthma group relative to non‐T2 controls may represent a more “experienced” pool of memory‐like ILC2 cells [27, 28] within those with non‐T2 asthma, but further studies in other cohorts are required to confirm this observation. Previous studies have observed that CD45RO+ ILC2s and CD8+ T memory cells are linked to steroid‐resistance [28, 29]. The enrichment of these cells in non‐T2 asthma suggests that they may contribute to persistent symptoms in patients who typically do not respond well to corticosteroids. Together, these findings suggest that CD45RO+ ILC2s and CD8+ T cells could serve as biomarkers for steroid‐unresponsive disease and may help identify patients who would benefit from alternative treatment strategies. This warrants consideration in future longitudinal and stratified studies aimed at improving care for non‐T2 asthma.

The non‐T2 endotype is challenging to capture since its definition relies on the absence of T2 features and is far less studied as compared to the T2 asthma endotype. Our definition of T2 features was based on specific IgE levels in serum for inhalant allergens and blood eosinophil count, which is in line with current literature [30]. The Global Initiative for Asthma (GINA) guidelines for defining T2 inflammation build upon a combination of increased sputum or blood eosinophils (with a lower cutoff in blood than what was used in this study), elevated FeNO, use of oral corticosteroids and/or clinically allergen‐driven features [31]. Our definition is based on available data from the BAMSE COVID‐19 follow‐up which did not include FeNO—however, addition of FeNO measured at the BAMSE 24‐year follow‐up to the subgroup definitions did not change the group composition notably, and the endotyping was reasonably stable over 1–5 years (Supporting Information S1: Figure S12). Also, few or no subjects in the asthma groups used oral corticosteroids. We acknowledge that the presence of IgE sensitization, rather than elevated blood eosinophils, was primarily differentiating T2 from non‐T2 asthma subjects in our study, and co‐occurrence of asthma and atopy is not necessarily the same as T2 asthma [32]. As is established, we defined the non‐T2 groups by an absence of the T2‐associated features [30]. A previous study on subjects of similar age as in BAMSE concluded that low/no measurable allergen‐specific IgE levels in serum are sufficient to identify non‐T2 asthma [33].

Cell populations typically associated with T2‐inflammation, namely ILC2s and CD4+ Th2 cells, were found to distinguish T2 asthma from non‐T2 asthma, supporting previous evidence that these cells contribute to this endotype [2]. Increased CRTH2+ CD4+ T cells, also called Th2 cells, associated with T2 features in both asthma and control subjects in our study, have previously been associated with asthma severity [34, 35]. T2 features are not exclusive to asthma but can also be detected in subjects without asthma with coexisting conditions that are driven by T2 inflammation. In line with previous literature, we also report increased prevalences of comorbidities typically characterized by T2 mechanisms—IgE sensitization, eczema, and rhinitis—in both the asthma and control groups with T2 features. This enabled the identification of lymphocytes specific to T2 features in asthma but not in controls, reflecting the real‐world overlap between T2 features and T2 comorbidities [36, 37, 38, 39]. Additionally, the groups with T2 features had higher leukocyte counts in peripheral blood, and it is attractive to hypothesize that T2 disease is a systemic disease, whereas the non‐T2 endotype is not well captured systemically (at least not in blood). Non‐T2 asthma has typically been linked to corticosteroid resistance and a more severe phenotype [30, 40], although T2 high signatures in severe asthma have been associated with remodeling and pronounced airway obstruction [41]. We did not observe, apart from lower FEV1 *z*‐scores in the non‐T2 asthma group, any differences in asthma severity or medication use between the asthma groups, albeit our findings may be limited by a small sample size, limited age span, and inclusion of subjects with mild‐moderate asthma.

We recognize that our study has some limitations. Firstly, the flow cytometric analysis was conducted on PBMCs, and immune cells from the airways were not collected. The target tissue was selected considering clinical feasibility and minimal invasiveness, yet this limits our understanding of the airway environment. Secondly, our flow cytometric assay was focused on selected surface markers primarily expressed by T2 subsets, thus limiting our ability to assess changes in regulatory, type 1, and 3 lymphocyte populations as well as intracellular markers and cytokines that may enhance the definition of cell populations and subsets [42]. Thirdly, we might have missed some relevant cell subpopulations due to the small sample size, especially within non‐T2 asthma. Lastly, our definition of T2 features was based on IgE levels measured 2 years prior to the time point when the eosinophils, ILCs, and T cells were measured in peripheral blood. In young adults, IgE levels are however not expected to change much over a 2‐year period [43]. Also, blood eosinophils were measured at a single time point, which constitutes a limitation as eosinophils are known to fluctuate over time; however, we additionally assessed blood eosinophil stability in our study population between the BAMSE 24‐year follow‐up and the COVID‐19 follow‐up, and observed relatively stable eosinophil levels between the two follow‐ups (data not shown) [44]. Additionally, FeNO was not included in our definition since this data was not available for the BAMSE COVID‐19 follow‐up.

This study has several strengths. The study design included well‐characterized subjects from the population‐based BAMSE cohort, including numerous asthma characteristics as well as lung function, BMI, and BF% measurements from the clinical examinations. Furthermore, a data‐driven approach was employed for dimensionality reduction and selection of the most relevant subpopulations. A final strength was the allocation of non‐asthma control subjects into T2 and non‐T2 groups, allowing us to capture T2 comorbidities in subjects without asthma and pinpoint subpopulations that are specific to the studied asthma endotypes.

## 5 Conclusions

In our study of young adults, T2 and non‐T2 asthma were found to be characterized by distinct immune profiles, respectively. Non‐T2 asthma was associated with a CD45RO+ ILC2 and CD8+ memory T cell signature, whereas T2 features with and without concurrent asthma were represented well by ILC2 and Th2 cell characteristics. We suggest that further investigation of ILC and CD8+ T cell subsets could offer a deeper understanding of particularly non‐T2 asthma.

## Author Contributions

**Maura M. Kere:** conceptualization, data curation, methodology, formal analysis, resources, writing – original draft. **Sophia Björkander:** conceptualization, data curation, methodology, project administration, resources, supervision, writing – review and editing. **Simon Kebede Merid:** methodology, formal analysis, writing – review and editing. **Natalia Hernandez‐Pacheco:** methodology, formal analysis, writing – review and editing. **Paul Maier:** data curation, investigation, resources, writing – review and editing. **Anne‐Sophie Merritt:** writing – review and editing, resources. **Anna Bergström:** resources, writing – review and editing. **Inger Kull:** resources, writing – review and editing. **Carsten O. Daub:** methodology, formal analysis, supervision, writing – review and editing. **Jenny Mjösberg:** conceptualization, funding acquisition, methodology, project administration, resources, supervision, writing – review and editing. **Christopher Andrew Tibbitt:** conceptualization, funding acquisition, data curation, investigation, methodology, formal analysis, project administration, resources, supervision, writing – review and editing. **Erik Mellén:** conceptualization, funding acquisition, methodology, project administration, resources, supervision, writing – review and editing.

## Ethics Statement

Informed written consent was obtained from all subjects and an ethical permit was granted by the Regional Ethics Review Board in Stockholm (nr: 2020‐02922 and 2024‐06052‐02).

## Consent

The authors have nothing to report.

## Conflicts of Interest

The authors declare no conflicts of interest.

## Supporting information


Supporting Information S1

## Data Availability

The datasets analyzed during the current study are not publicly available since the authors elect not to share data but are available from the corresponding author on reasonable request.
