# Supplementary material for: Distinct Phenotypes of Peripheral Innate Lymphoid Cells and T Cells in Type 2 and Non‐Type 2 Asthma
Source: Clin Transl Allergy. 2025 Sep 18;15(9):e70108. doi: 10.1002/clt2.70108 (PMC12444775; doi:10.1002/clt2.70108)
Supplement: Supplementary file 1 — Supporting Information S1 [file CLT2-15-e70108-s001.docx]

**Supplementary Table S1.** Distribution of type 2 features in groups with and without asthma.

|  | **Type 2 asthma (n=30)** | **Type 2 control (n=20)** |
| --- | --- | --- |
| High blood eosinophil count only (≥ 0.3x10^9^/L), n (%) | 2 (6.7) | 7 (35.0) |
| IgE-sensitization to inhalant allergens only (≥0.35 kU/l), n (%) | 20 (66.7) | 12 (60.0) |
| Both high blood eosinophil count and IgE-sensitization to inhalant allergens, n (%) | 8 (26.7) | 1 (5.0) |

**Supplementary Table S2**. Asthma severity markers in subjects with asthma (n=40). Variables are presented as n (%). If the answer frequency was <100%, the total number of answers is indicated after “/”.

|  | **Type 2 asthma (n= 30)** | **Non-type 2 asthma (n=10)** | **p-value** |
| --- | --- | --- | --- |
| Any breathing difficulties ^A^ | 18 (60.0) | 6 (60.0) | 1.000 ^B^ |
| Activity limitation due to breathing difficulties ^A^ | 6/18 (33.3) | 4/6 (66.7) | 0.192 ^B^ |
| Nocturnal awakening(s) due to breathing difficulties ^A^ | 5/18 (27.8) | 0/6 (0.0) | 0.280 ^B^ |
| Use of any medication for asthma or breathing difficulties ^A^ | 19 (63.3) | 5 (50.0) | 0.482 ^B^ |
| Use of any inhaled steroids for asthma or breathing difficulties ^A^ | 9/9 (100.0) | 3/3 (100.0) | NA |
| Use of oral steroids for asthma or breathing difficulties ^A^ | 1/19 (5.3) | 0/5 (0.0) | 1.000 ^B^ |
| Emergency department visit due to asthma or breathing difficulties ^A^ | 1 (3.3) | 0 (0.0) | 1.000 ^B^ |
| Hospitalization due to asthma or breathing difficulties ^A^ | 0 (0.0) | 0 (0.0) | NA |

^A^=in the past 2-9 months (since August 1^st^, 2020), ^B^=Fisher’s exact test.

**Supplementary Table S3.** Longitudinal trends of asthma and allergic comorbidities in the BAMSE cohort. Variables are presented as n (%). If the answer frequency was <100%, the total number of answers is indicated after “/” (or as missing data). All p-values were determined using Fisher’s exact test.

|  | **Type 2 asthma (n=30)** | **Non-type 2 asthma (n=10)** | **Type 2 control (n=20)** | **Non-type 2 control (n=26)** | **p-value** |
| --- | --- | --- | --- | --- | --- |
| **Heredity for asthma and atopy^1^** | 14/29 (48.3) | 4/9 (44.4) | 10/19 (52.6) | 6 (23.1) | 0.158 |
| **Asthma over time^2^** | | | | | **0.006** |
| No asthma at any timepoint | 6/26 (23.1) | 1 (10.0) | 8/14 (57.1) | 16/22 (72.7) |  |
| Early-onset asthma (at age 1, 2 or 4 years of age) | 3/26 (11.5) | 1 (10.0) | 3/14 (21.4) | 2/22 (9.1) |  |
| School-age onset (at 8, 12 or 16 years of age) | 8/26 (30.8) | 3 (30.0) | 2/14 (14.3) | 3/22 (13.6) |  |
| Persistent asthma up to 16 years of age (at 1, 2 or 4 and at 8, 12 or 16 years of age) | 9/26 (34.6) | 5 (50.0) | 1/14 (7.1) | 1/22 (4.5) |  |
| **Eczema over time^3^** | | | | | 0.281 |
| No eczema at any timepoint | 9/26 (34.6) | 7/9 (77.8) | 7/14 (50.0) | 15/21 (71.4) |  |
| Early onset/transient (eczema up to 4 years of age) | 3/26 (11.5) | 0/9 (0.0) | 1/14 (7.1) | 2/21 (9.5) |  |
| Late-onset/school-age onset (eczema onset from 7 to 16 years of age) | 3/26 (11.5) | 1/9 (11.1) | 2/14 (14.3) | 2/21 (9.5) |  |
| Persistent asthma (up to 4 years and between 7 and 16 years of age) | 11/26 (42.3) | 1/9 (11.1) | 4/14 (28.6) | 2/21 (9.5) |  |
| **Rhinitis over time^4^** | | | | | **0.002** |
| No rhinitis at any timepoint | 5/27 (18.5) | 8 (80.0) | 5/13 (38.5) | 18/21 (85.7) |  |
| Early-onset/transient (onset up to 4 years of age) | 3/27 (11.1) | 0 (0.0) | 1/13 (7.7) | 1/21 (4.8) |  |
| Late-onset/school age (onset from 7 years up to 16 years of age) | 12/27 (44.4) | 1 (10.0) | 5/13 (38.5) | 2/21 (9.5) |  |
| Persistent rhinitis (up to 4 years and between 7 and 16 years of age) | 7/27 (25.9) | 1 (10.0) | 2/13 (15.4) | 0/21 (0.0) |  |
| **IgE-sensitization to inhalant allergens at** | | | | |  |
| Age 4 years | 9/24 (37.5) | 0/7 (0.0) | 2/17 (11.8) | 0/21 (0.0) | **0.003** |
| Age 8 years | 13/25 (52.0) | 0/8 (0.0) | 5/14 (35.7) | 0/22 (0.0) | **<0.001** |
| Age 16 years | 21/28 (75.0) | 1 (10.0) | 10/15 (66.7) | 2/23 (8.7) | **<0.001** |
| **IgE-sensitization to food allergens at** | | | | |  |
| Age 4 years | 11/24 (45.8) | 2/7 (28.6) | 1/17 (5.9) | 1/21 (4.8) | **0.002** |
| Age 8 years | 12/25 (48.0) | 1/8 (12.5) | 4/14 (28.6) | 0/22 (0.0) | **0.002** |
| Age 16 years | 9/28 (32.1) | 3 (30.0) | 4/15 (26.7) | 0/23 (0.0) | **0.030** |

^1^ **=** Mother and/or father with doctor’s diagnosis of asthma and asthma medication and/or doctor’s diagnosis of hay fever in combination with

pet or pollen allergy at baseline.

^2^ = up to 16 years of age. At least 2/3 criteria: doctor’s diagnosis of asthma up to the age of 16 years, symptoms of breathing difficulties and/or asthma medication use occasionally or regularly in the last 12 months.

^3^ =up to 16 years of age. Dry skin in combination with itchy rash for 2 weeks or more and typical localization (face or arms/legs extension surfaces or arms/legs flexures or wrists/ankles flexures) in the last 12 months prior and/or doctor’s diagnosis of eczema up to the age of 16 years.

^4^= up to 16 years of age. Symptoms of sneezing, a runny or blocked nose, or itchy, red and watery eyes after exposure to furred pets or pollen in the last 12 months and/or doctor's diagnosis of allergic rhinitis up to the age of 16 years.

**Supplementary Table S4**. The number of clusters per cell type (ILC/NK cells, CD4 T cells, CD8 T cells) and the number of cell subpopulations in each cluster after hierarchical clustering.

| **ILC/NK cells** | | | | | | | | | | | | | | | | |
| --- | --- | --- | --- | --- | --- | --- | --- | --- | --- | --- | --- | --- | --- | --- | --- | --- |
| **ILC/NK cluster** | **1** | **2** | **3** | **4** | **5** | **6** | **7** | **8** | **9** | **10** | **11** | **12** | **13** | **14** | **15** | **16** |
| **Number of subpopulations** | 12 | 13 | 11 | 11 | 8 | 12 | 10 | 11 | 4 | 27 | 6 | 12 | 11 | 8 | 6 | 4 |
| **CD4 T cells** | | | | | | | | | | | | | | | | |
| **CD4 cluster** | **1** | **2** | **3** | **4** | **5** | **6** | **7** | **8** | **9** | **10** | **11** | **12** | **13** | **14** | **15** | **16** |
| **Number of subpopulations** | 5 | 6 | 4 | 6 | 7 | 11 | 6 | 4 | 12 | 6 | 11 | 8 | 8 | 6 | 12 | 10 |
| **CD8 T cells** | | | | | | | | | | | | | | | | |
| **CD8 cluster** | **1** | **2** | **3** | **4** | **5** | **6** | **7** | **8** | **9** | **10** | **11** | **12** | **13** | **14** | **15** | **16** |
| **Number of subpopulations** | 4 | 9 | 10 | 17 | 12 | 8 | 8 | 16 | 8 | 14 | 8 | 8 | - | - | - | - |

**Supplementary Table S5**. ILC/NK cluster 5, CD4 cluster 16, and CD4 cluster 9 representative variables (RVs) are associated with type 2 (T2) asthma (comparison set 1) and presented in Table 2 (bolded here). Full results from a logistic regression model: RVs as predictors, T2 asthma as the outcome, non-T2 asthma as the reference group. Adjusted for sex, BMI, smoking, and blood lymphocyte count.

| **Cluster** | **Odds ratio [95% confidence interval]** | **P value** |
| --- | --- | --- |
| **ILC/NK Cluster 5 RV** | **4.24 [1.21, 14.82]** | **0.02** |
| ILC/NK Cluster 6 RV | 3.07 [0.81, 11.56] | 0.10 |
| ILC/NK Cluster 9 RV | 0.48 [0.19, 1.25] | 0.13 |
| ILC/NK Cluster 7 RV | 0.52 [0.20, 1.33] | 0.17 |
| ILC/NK Cluster 15 RV | 1.64 [0.58, 4.61] | 0.35 |
| ILC/NK Cluster 3 RV | 1.59 [0.52, 4.83] | 0.42 |
| ILC/NK Cluster 2 RV | 0.70 [0.25, 1.92] | 0.48 |
| ILC/NK Cluster 14 RV | 0.65 [0.18, 2.27] | 0.50 |
| ILC/NK Cluster 4 RV | 0.71 [0.21, 2.37] | 0.58 |
| ILC/NK Cluster 13 RV | 0.77 [0.26, 2.30] | 0.64 |
| ILC/NK Cluster 16 RV | 1.12 [0.48, 2.59] | 0.79 |
| ILC/NK Cluster 1 RV | 0.88 [0.16, 4.85] | 0.89 |
| ILC/NK Cluster 11 RV | 0.95 [0.41, 2.20] | 0.91 |
| ILC/NK Cluster 12 RV | 1.05 [0.28, 3.98] | 0.94 |
| ILC/NK Cluster 10 RV | 1.03 [0.22, 4.93] | 0.97 |
| ILC/NK Cluster 8 RV | 1.00 [0.28, 3.57] | 1.00 |
| **CD4 Cluster 16 RV** | **6.63 [1.56, 28.20]** | **0.01** |
| **CD4 Cluster 9 RV** | **0.28 [0.09, 0.89]** | **0.03** |
| CD4 Cluster 6 RV | 3.03 [0.88, 10.42] | 0.08 |
| CD4 Cluster 3 RV | 2.10 [0.89, 4.97] | 0.09 |
| CD4 Cluster 8 RV | 2.73 [0.80, 9.34] | 0.11 |
| CD4 Cluster 15 RV | 0.33 [0.08, 1.32] | 0.12 |
| CD4 Cluster 12 RV | 0.51 [0.22, 1.21] | 0.13 |
| CD4 Cluster 13 RV | 0.52 [0.23, 1.21] | 0.13 |
| CD4 Cluster 5 RV | 0.53 [0.22, 1.30] | 0.17 |
| CD4 Cluster 7 RV | 1.40 [0.66, 2.97] | 0.39 |
| CD4 Cluster 11 RV | 0.58 [0.16, 2.11] | 0.41 |
| CD4 Cluster 2 RV | 0.70 [0.27, 1.83] | 0.46 |
| CD4 Cluster 1 RV | 1.46 [0.53, 3.99] | 0.46 |
| CD4 Cluster 14 RV | 0.74 [0.32, 1.70] | 0.48 |
| CD4 Cluster 10 RV | 0.81 [0.28, 2.37] | 0.71 |
| CD4 Cluster 4 RV | 0.90 [0.37, 2.19] | 0.81 |
| CD8 Cluster 11 RV | 0.33 [0.11, 1.02] | 0.05 |
| CD8 Cluster 5 RV | 2.25 [0.79, 6.44] | 0.13 |
| CD8 Cluster 3 RV | 0.50 [0.19, 1.30] | 0.16 |
| CD8 Cluster 1 RV | 1.53 [0.69, 3.42] | 0.30 |
| CD8 Cluster 2 RV | 0.68 [0.25, 1.87] | 0.46 |
| CD8 Cluster 6 RV | 0.68 [0.21, 2.24] | 0.53 |
| CD8 Cluster 8 RV | 0.62 [0.13, 3.02] | 0.55 |
| CD8 Cluster 7 RV | 1.36 [0.42, 4.38] | 0.61 |
| CD8 Cluster 12 RV | 0.81 [0.36, 1.84] | 0.62 |
| CD8 Cluster 4 RV | 0.85 [0.26, 2.79] | 0.78 |
| CD8 Cluster 9 RV | 0.94 [0.40, 2.22] | 0.89 |
| CD8 Cluster 10 RV | 0.94 [0.39, 2.26] | 0.90 |

**Supplementary Table S6.** ILC/NK cluster 3 and CD4 cluster 16 representative variables (RVs) are associated with type 2 (T2) controls (comparison set 2) and presented in Table 2 (bolded here). Full results from a logistic regression model: RVs as predictors, T2 control as the outcome, non-T2 control as the reference group. Adjusted for sex, BMI, smoking, and blood lymphocyte count.

| **Cluster** | **Odds ratio [95% confidence interval]** | **P value** |
| --- | --- | --- |
| **ILC/NK Cluster 3 RV** | **4.86 [1.36, 17.39]** | **0.02** |
| ILC/NK Cluster 6 RV | 1.90 [0.73, 4.96] | 0.19 |
| ILC/NK Cluster 11 RV | 1.68 [0.76, 3.68] | 0.20 |
| ILC/NK Cluster 13 RV | 0.52 [0.18, 1.51] | 0.23 |
| ILC/NK Cluster 16 RV | 1.46 [0.70, 3.03] | 0.31 |
| ILC/NK Cluster 10 RV | 2.15 [0.47, 9.83] | 0.32 |
| ILC/NK Cluster 4 RV | 0.55 [0.17, 1.81] | 0.33 |
| ILC/NK Cluster 12 RV | 0.60 [0.21, 1.69] | 0.34 |
| ILC/NK Cluster 9 RV | 0.80 [0.36, 1.80] | 0.59 |
| ILC/NK Cluster 1 RV | 0.86 [0.24, 3.00] | 0.81 |
| ILC/NK Cluster 7 RV | 0.91 [0.36, 2.27] | 0.83 |
| ILC/NK Cluster 5 RV | 0.93 [0.41, 2.11] | 0.86 |
| ILC/NK Cluster 2 RV | 1.06 [0.38, 2.96] | 0.92 |
| ILC/NK Cluster 14 RV | 0.94 [0.33, 2.74] | 0.92 |
| ILC/NK Cluster 15 RV | 1.02 [0.47, 2.23] | 0.95 |
| ILC/NK Cluster 8 RV | 0.97 [0.31, 3.01] | 0.96 |
| **CD4 Cluster 16 RV** | **2.19 [1.06, 4.50]** | **0.03** |
| CD4 Cluster 7 RV | 1.91 [0.89, 4.08] | 0.10 |
| CD4 Cluster 11 RV | 2.29 [0.86, 6.10] | 0.10 |
| CD4 Cluster 5 RV | 0.58 [0.25, 1.31] | 0.19 |
| CD4 Cluster 9 RV | 0.65 [0.30, 1.40] | 0.27 |
| CD4 Cluster 13 RV | 0.64 [0.28, 1.45] | 0.28 |
| CD4 Cluster 1 RV | 0.60 [0.21, 1.65] | 0.32 |
| CD4 Cluster 10 RV | 0.74 [0.37, 1.49] | 0.41 |
| CD4 Cluster 4 RV | 0.71 [0.31, 1.64] | 0.42 |
| CD4 Cluster 12 RV | 0.67 [0.23, 1.93] | 0.46 |
| CD4 Cluster 2 RV | 1.46 [0.51, 4.13] | 0.48 |
| CD4 Cluster 6 RV | 1.28 [0.53, 3.08] | 0.59 |
| CD4 Cluster 8 RV | 1.21 [0.53, 2.76] | 0.65 |
| CD4 Cluster 15 RV | 1.18 [0.53, 2.61] | 0.69 |
| CD4 Cluster 14 RV | 1.13 [0.52, 2.45] | 0.76 |
| CD4 Cluster 3 RV | 1.11 [0.46, 2.66] | 0.81 |
| CD8 Cluster 6 RV | 2.71 [0.99, 7.40] | 0.05 |
| CD8 Cluster 2 RV | 2.16 [0.80, 5.84] | 0.13 |
| CD8 Cluster 4 RV | 0.47 [0.16, 1.38] | 0.17 |
| CD8 Cluster 9 RV | 0.54 [0.21, 1.41] | 0.21 |
| CD8 Cluster 10 RV | 1.61 [0.71, 3.63] | 0.25 |
| CD8 Cluster 3 RV | 0.56 [0.21, 1.51] | 0.25 |
| CD8 Cluster 5 RV | 1.54 [0.68, 3.51] | 0.30 |
| CD8 Cluster 12 RV | 0.70 [0.33, 1.47] | 0.35 |
| CD8 Cluster 11 RV | 0.75 [0.36, 1.57] | 0.44 |
| CD8 Cluster 7 RV | 1.25 [0.52, 3.03] | 0.61 |
| CD8 Cluster 8 RV | 1.23 [0.46, 3.30] | 0.68 |
| CD8 Cluster 1 RV | 1.04 [0.45, 2.41] | 0.93 |

**Supplementary Table S7.** CD8 cluster 6 representative variable (RV) is associated with type 2 (T2) asthma (comparison set 3) and presented in Table 2 (bolded here). Full results from a logistic regression model: RVs as predictors, T2 asthma as the outcome, T2 control as the reference group. Adjusted for sex, BMI, smoking, and blood lymphocyte count.

| **Cluster** | **Odds ratio [95% confidence interval]** | **P value** |
| --- | --- | --- |
| ILC/NK Cluster 5 RV | 2.04 [0.94, 4.44] | 0.07 |
| ILC/NK Cluster 2 RV | 0.50 [0.21, 1.24] | 0.14 |
| ILC/NK Cluster 14 RV | 0.46 [0.16, 1.36] | 0.16 |
| ILC/NK Cluster 11 RV | 0.63 [0.33, 1.21] | 0.17 |
| ILC/NK Cluster 10 RV | 0.52 [0.15, 1.88] | 0.32 |
| ILC/NK Cluster 15 RV | 0.70 [0.33, 1.49] | 0.36 |
| ILC/NK Cluster 3 RV | 0.68 [0.29, 1.58] | 0.37 |
| ILC/NK Cluster 13 RV | 1.41 [0.61, 3.25] | 0.42 |
| ILC/NK Cluster 7 RV | 1.31 [0.62, 2.79] | 0.48 |
| ILC/NK Cluster 1 RV | 1.45 [0.44, 4.76] | 0.54 |
| ILC/NK Cluster 9 RV | 0.80 [0.38, 1.65] | 0.54 |
| ILC/NK Cluster 12 RV | 1.22 [0.47, 3.13] | 0.68 |
| ILC/NK Cluster 8 RV | 1.15 [0.36, 3.64] | 0.81 |
| ILC/NK Cluster 6 RV | 1.04 [0.47, 2.27] | 0.93 |
| ILC/NK Cluster 4 RV | 0.98 [0.32, 3.01] | 0.97 |
| ILC/NK Cluster 16 RV | 1.00 [0.54, 1.86] | 0.99 |
| CD4 Cluster 8 RV | 1.99 [0.76, 5.19] | 0.16 |
| CD4 Cluster 3 RV | 1.41 [0.69, 2.91] | 0.35 |
| CD4 Cluster 9 RV | 0.71 [0.34, 1.50] | 0.37 |
| CD4 Cluster 5 RV | 1.35 [0.68, 2.68] | 0.39 |
| CD4 Cluster 7 RV | 0.73 [0.35, 1.52] | 0.40 |
| CD4 Cluster 1 RV | 1.33 [0.64, 2.77] | 0.44 |
| CD4 Cluster 15 RV | 0.76 [0.35, 1.62] | 0.47 |
| CD4 Cluster 12 RV | 0.78 [0.39, 1.56] | 0.48 |
| CD4 Cluster 14 RV | 1.28 [0.64, 2.55] | 0.49 |
| CD4 Cluster 10 RV | 0.77 [0.35, 1.67] | 0.51 |
| CD4 Cluster 16 RV | 1.27 [0.56, 2.88] | 0.56 |
| CD4 Cluster 2 RV | 0.82 [0.34, 1.97] | 0.66 |
| CD4 Cluster 4 RV | 1.17 [0.47, 2.91] | 0.74 |
| CD4 Cluster 13 RV | 0.89 [0.45, 1.75] | 0.74 |
| CD4 Cluster 11 RV | 0.93 [0.38, 2.32] | 0.88 |
| CD4 Cluster 6 RV | 1.06 [0.46, 2.47] | 0.89 |
| **CD8 Cluster 6** **RV** | **0.35 [0.13, 0.97]** | **0.04** |
| CD8 Cluster 4 RV | 2.10 [0.68, 6.47] | 0.20 |
| CD8 Cluster 10 RV | 0.74 [0.36, 1.50] | 0.40 |
| CD8 Cluster 11 RV | 0.74 [0.35, 1.54] | 0.42 |
| CD8 Cluster 3 RV | 0.78 [0.37, 1.62] | 0.50 |
| CD8 Cluster 9 RV | 1.29 [0.60, 2.76] | 0.52 |
| CD8 Cluster 2 RV | 1.25 [0.53, 2.96] | 0.62 |
| CD8 Cluster 1 RV | 1.17 [0.58, 2.36] | 0.65 |
| CD8 Cluster 8 RV | 1.19 [0.43, 3.34] | 0.74 |
| CD8 Cluster 7 RV | 0.87 [0.35, 2.14] | 0.76 |
| CD8 Cluster 5 RV | 0.90 [0.40, 2.02] | 0.79 |
| CD8 Cluster 12 RV | 1.06 [0.54, 2.05] | 0.87 |

**Supplementary Table S8.** ILC/NK cluster 7, CD4 cluster 14, and CD8 cluster 2 representative variables (RVs) are associated with non-type 2 (non-T2) asthma and presented in Table 2 (bolded here). Full results from a logistic regression model: RVs as predictors, non-T2 asthma as outcome, non-T2 control as reference group. Adjusted for sex, BMI, smoking, and blood lymphocyte count.

| **Cluster** | **Odds ratio [95% confidence interval]** | **P value** |
| --- | --- | --- |
| **ILC/NK Cluster 7 RV** | **5.71 [1.15, 28.34]** | **0.03** |
| ILC/NK Cluster 5 RV | 0.36 [0.10, 1.28] | 0.11 |
| ILC/NK Cluster 15 RV | 0.39 [0.10, 1.58] | 0.19 |
| ILC/NK Cluster 16 RV | 2.10 [0.66, 6.71] | 0.21 |
| ILC/NK Cluster 9 RV | 1.88 [0.64, 5.50] | 0.25 |
| ILC/NK Cluster 1 RV | 2.81 [0.39, 20.18] | 0.30 |
| ILC/NK Cluster 13 RV | 2.08 [0.48, 9.01] | 0.33 |
| ILC/NK Cluster 6 RV | 0.62 [0.20, 1.91] | 0.41 |
| ILC/NK Cluster 10 RV | 1.89 [0.27, 13.30] | 0.52 |
| ILC/NK Cluster 14 RV | 1.47 [0.39, 5.52] | 0.57 |
| ILC/NK Cluster 3 RV | 1.50 [0.33, 6.87] | 0.60 |
| ILC/NK Cluster 11 RV | 0.90 [0.33, 2.43] | 0.84 |
| ILC/NK Cluster 8 RV | 0.89 [0.23, 3.42] | 0.87 |
| ILC/NK Cluster 2 RV | 0.96 [0.31, 2.97] | 0.95 |
| ILC/NK Cluster 4 RV | 0.98 [0.28, 3.37] | 0.97 |
| ILC/NK Cluster 12 RV | 0.99 [0.25, 3.90] | 0.99 |
| **CD4 Cluster 14 RV** | **3.06 [1.02, 9.22]** | **0.05** |
| CD4 Cluster 11 RV | 4.47 [0.87, 23.04] | 0.07 |
| CD4 Cluster 15 RV | 2.84 [0.87, 9.26] | 0.08 |
| CD4 Cluster 5 RV | 2.13 [0.70, 6.44] | 0.18 |
| CD4 Cluster 2 RV | 1.69 [0.58, 4.96] | 0.34 |
| CD4 Cluster 10 RV | 0.65 [0.26, 1.65] | 0.37 |
| CD4 Cluster 3 RV | 0.67 [0.26, 1.70] | 0.40 |
| CD4 Cluster 6 RV | 0.64 [0.23, 1.81] | 0.40 |
| CD4 Cluster 12 RV | 1.54 [0.55, 4.32] | 0.41 |
| CD4 Cluster 13 RV | 1.46 [0.60, 3.57] | 0.41 |
| CD4 Cluster 1 RV | 0.60 [0.17, 2.14] | 0.43 |
| CD4 Cluster 9 RV | 1.41 [0.49, 4.10] | 0.52 |
| CD4 Cluster 16 RV | 1.29 [0.53, 3.13] | 0.58 |
| CD4 Cluster 4 RV | 0.83 [0.38, 1.83] | 0.65 |
| CD4 Cluster 7 RV | 1.13 [0.52, 2.46] | 0.76 |
| CD4 Cluster 8 RV | 1.16 [0.41, 3.31] | 0.78 |
| **CD8 Cluster 2** **RV** | **3.71 [1.06, 13.04]** | **0.04** |
| CD8 Cluster 11 RV | 2.42 [0.81, 7.28] | 0.12 |
| CD8 Cluster 6 RV | 2.32 [0.64, 8.41] | 0.20 |
| CD8 Cluster 1 RV | 0.61 [0.23, 1.65] | 0.33 |
| CD8 Cluster 5 RV | 0.60 [0.21, 1.75] | 0.35 |
| CD8 Cluster 8 RV | 1.59 [0.45, 5.62] | 0.47 |
| CD8 Cluster 12 RV | 1.18 [0.47, 2.92] | 0.73 |
| CD8 Cluster 7 RV | 0.87 [0.28, 2.70] | 0.81 |
| CD8 Cluster 3 RV | 0.87 [0.29, 2.66] | 0.81 |
| CD8 Cluster 4 RV | 1.11 [0.35, 3.52] | 0.85 |
| CD8 Cluster 9 RV | 1.07 [0.35, 3.29] | 0.90 |
| CD8 Cluster 10 RV | 0.97 [0.40, 2.35] | 0.95 |

**Supplementary Table S9**. ILC/NK subpopulations divided into clusters after hierarchical clustering; only relevant clusters shown. **Not included in supplementary figures. **renamed for Figure 1 or 2; original subpopulation name shown in the Supplementary figure.*

| **ILC/NK cluster** | **Subpopulation group** | **Subpopulation name** |
| --- | --- | --- |
| 3 | ILC % of parent | CD117+ ILC2 \| Freq. of Parent [%] |
| 3 | ILC % of parent | CD117- ILC2 \| Freq. of Parent [%] |
| 3 | ILC % of parent | CD117- ILC \| Geometric Mean (CD45RA AF700) |
| 3 | ILC % of parent | CD117- ILC \| Geometric Mean (CD62L APC Cy7) |
| 3 | ILC % of parent | CD117- ILC \| Geometric Mean (CD200R PB) |
| *3* | *ILC % of parent* | *CD117- ILC \| Geometric Mean (CRTH2 PE CF594)** |
| 3 | ILC % of parent | CD117- ILC \| Geometric Mean (CD117 PE Cy55) |
| 3 | ILC % of parent | CD117- CD45RA+ ILC \| Freq. of Parent |
| 3 | ILC % of parent | CD117- CD62L+ ILC \| Freq. of Parent |
| 3 | ILC % of parent | ILC2 \| Freq. of Parent |
| *3* | *ILC % of parent* | *ILCp \| Geometric Mean (CRTH2 PE CF594)** |
| 5 | ILC % of parent | CD117+ ILC2 \| Geometric Mean (CD62L APC Cy7) |
| 5 | ILC % of parent | CD117+ CD62L+ ILC2 \| Freq. of Parent |
| 5 | ILC % of parent | CD117- ILC2 \| Geometric Mean (CD62L APC Cy7) |
| 5 | ILC % of parent | CD117- CD62L+ ILC2 \| Freq. of Parent |
| 5 | ILC % of parent | ILC2 \| Geometric Mean (CD62L APC Cy7) |
| 5 | ILC % of parent | CD62L+ ILC2 \| Freq. of Parent (renamed: % CD62L+ of ILC2)** |
| 5 | ILC % of parent | ILCp \| Geometric Mean (CD62L APC Cy7) |
| 5 | ILC % of parent | CD62L+ ILCp \| Freq. of Parent |
| *7* | *ILC % of parent* | *CD117+ ILC2 \| Geometric Mean (CD45RO BV650)** |
| 7 | ILC % of parent | CD117+ CD45RO+ ILC2 \| Freq. of Parent |
| *7* | *ILC % of parent* | *CD117- ILC2 \| Geometric Mean (CD45RO BV650)** |
| *7* | *ILC % of parent* | *CD117- ILC2 \| Geometric Mean (CD4 BV785)** |
| 7 | ILC % of parent | CD117- CD4+ ILC2 \| Freq. of Parent |
| 7 | ILC % of parent | CD117- CD45RO+ ILC2 \| Freq. of Parent |
| *7* | *ILC % of parent* | *ILC2 \| Geometric Mean (CD45RO BV650)** |
| 7 | ILC % of parent | ILC2 \| Geometric Mean (CD4 BV785) |
| 7 | ILC % of parent | CD4+ ILC2 \| Freq. of Parent |
| 7 | ILC % of parent | CD45RO+ ILC2 \| Freq. of Parent (renamed: % CD45RO+ of ILC2)** |

**Supplementary Table S10**. CD4 subpopulations divided into clusters after hierarchical clustering; only relevant clusters shown. **renamed for Figure 1 or 2; original subpopulation name shown in the Supplementary figure.*

| **CD4 cluster** | **Subpopulation group** | **Subpopulation name** |
| --- | --- | --- |
| 9 | CD4+ % of parent | CD4+ naive \| Geometric Mean (KLRG1 PE Cy7) |
| 9 | CD4+ % of parent | CD4+ KLRG1+ naive \| Freq. of Parent |
| 9 | CD4+ % of parent | CD4+ TCM \| Geometric Mean (KLRG1 PE Cy7) |
| 9 | CD4+ % of parent | CD4+ TCM \| Geometric Mean (KLRG1 PE Cy7) |
| 9 | CD4+ % of parent | CD4+ KLRG1+ TCM \| Freq. of Parent (renamed: KLRG1+ of CD4+ TCM)* |
| 9 | CD4+ % of parent | CD4+ KLRG1+ TCM \| Freq. of Parent |
| 9 | CD4+ % of parent | CD4+ TEM \| Geometric Mean (KLRG1 PE Cy7) |
| 9 | CD4+ % of parent | CD4+ TEM \| Geometric Mean (KLRG1 PE Cy7) |
| 9 | CD4+ % of parent | CD4+ KLRG1+ TEM \| Freq. of Parent |
| 9 | CD4+ % of parent | CD4+ KLRG1+ TEM \| Freq. of Parent |
| 9 | CD4+ % of parent | CD4+ TEMRA \| Geometric Mean (KLRG1 PE Cy7) |
| 9 | CD4+ % of parent | CD4+ KLRG1+ TEMRA \| Freq. of Parent |
| 14 | CD4+ % of parent | CD4+ TCM \| Geometric Mean (CD45RO BV650) |
| 14 | CD4+ % of parent | CD4+ CD45RO+ TCM \| Freq. of Parent |
| 14 | CD4+ % of parent | CD4+ CD45RO+ TCM \| Geometric Mean (CD45RO BV650) |
| 14 | CD4+ % of parent | CD4+ TEM \| Geometric Mean (CD45RO BV650) |
| 14 | CD4+ % of parent | CD4+ CD45RO+ TEM \| Freq. of Parent |
| 14 | CD4+ % of parent | CD4+ CD45RO+ TEM \| Geometric Mean (CD45RO BV650) |
| 16 | CD4+ % of parent | CD4+ CD200R+ CRTH2+ TCM \| Freq. of Parent |
| 16 | CD4+ % of parent | CD4+ CD200R- CRTH2+ TCM \| Freq. of Parent |
| 16 | CD4+ % of parent | CD4+ CRTH2+ TCM \| Freq. of Parent (renamed: % CRTH2+ of CD4+ TCM)* |
| 16 | CD4+ % of parent | CD4+ CD200R+ CRTH2+ TCM \| Freq. of Parent |
| 16 | CD4+ % of parent | CD4+ CD200R- CRTH2+ TCM \| Freq. of Parent |
| 16 | CD4+ % of parent | CD4+ CRTH2+ TCM \| Freq. of Parent |
| 16 | CD4+ % of parent | CD4+ CD200R+ CRTH2+ TEM \| Freq. of Parent |
| 16 | CD4+ % of parent | CD4+ CRTH2+ TCM \| Freq. of Parent |
| 16 | CD4+ % of parent | CD4+ CD200R+ CRTH2+ TEM \| Freq. of Parent |
| 16 | CD4+ % of parent | CD4+ CRTH2+ TEM \| Freq. of Parent (renamed: % CRTH2+ of CD4+ TEM)* |

**Supplementary Table S11**. CD8 subpopulations divided into clusters after hierarchical clustering; only relevant clusters shown. **renamed for Figure 1 or 2; original subpopulation name shown in the Supplementary figure.*

| **CD8 cluster** | **Subpopulation group** | **Subpopulation name** |
| --- | --- | --- |
| 2 | CD8+ % of parent | CD8+ naive \| Geometric Mean (CD45RA AF700) |
| 2 | CD8+ % of parent | CD8+ TCM \| Geometric Mean (CD45RO BV650) |
| 2 | CD8+ % of parent | CD8+ CD45RO+ TCM \| Freq. of Parent (renamed: % CD45RO+ of CD8+ TCM)* |
| 2 | CD8+ % of parent | CD8+ CD45RO+ TCM \| Geometric Mean (CD45RO BV650) |
| 2 | CD8+ % of parent | CD8+ TEM \| Geometric Mean (CD45RO BV650) |
| 2 | CD8+ % of parent | CD8+ CD45RO+ TEM \| Freq. of Parent (renamed: % CD45RO+ of CD8+ TEM)* |
| 2 | CD8+ % of parent | CD8+ CD45RO+ TEM \| Geometric Mean (CD45RO BV650) |
| 2 | CD8+ % of parent | CD8+ TEM \| Freq. of Parent |
| 2 | CD8+ % of parent | CD8+ TEM \| Geometric Mean (CD45RA AF700) |
| 6 | CD8+ % of parent | CD8+ naive \| Geometric Mean (CRTH2 PE CF594) |
| 6 | CD8+ % of parent | CD8+ CD117+ naive \| Freq. of Parent |
| 6 | CD8+ % of parent | CD8+ CD117+ TCM \| Freq. of Parent |
| 6 | CD8+ % of parent | CD8+ CD117+ TCM \| Freq. of Parent |
| 6 | CD8+ % of parent | CD8+ CD117+ TEM \| Freq. of Parent |
| 6 | CD8+ % of parent | CD8+ CD117+ TEM \| Freq. of Parent |
| 6 | CD8+ % of parent | CD8+ TEMRA \| Geometric Mean (CRTH2 PE CF594) |
| 6 | CD8+ % of parent | CD8+ CD117+ TEMRA \| Freq. of Parent |

**Supplementary Figure S1.** Set-up of the first 2 phases of the BAMSE COVID-19 follow-up and study subject selection. BF%= body fat percentage, BMI= body mass index, COVID-19: coronavirus disease 2019.

**
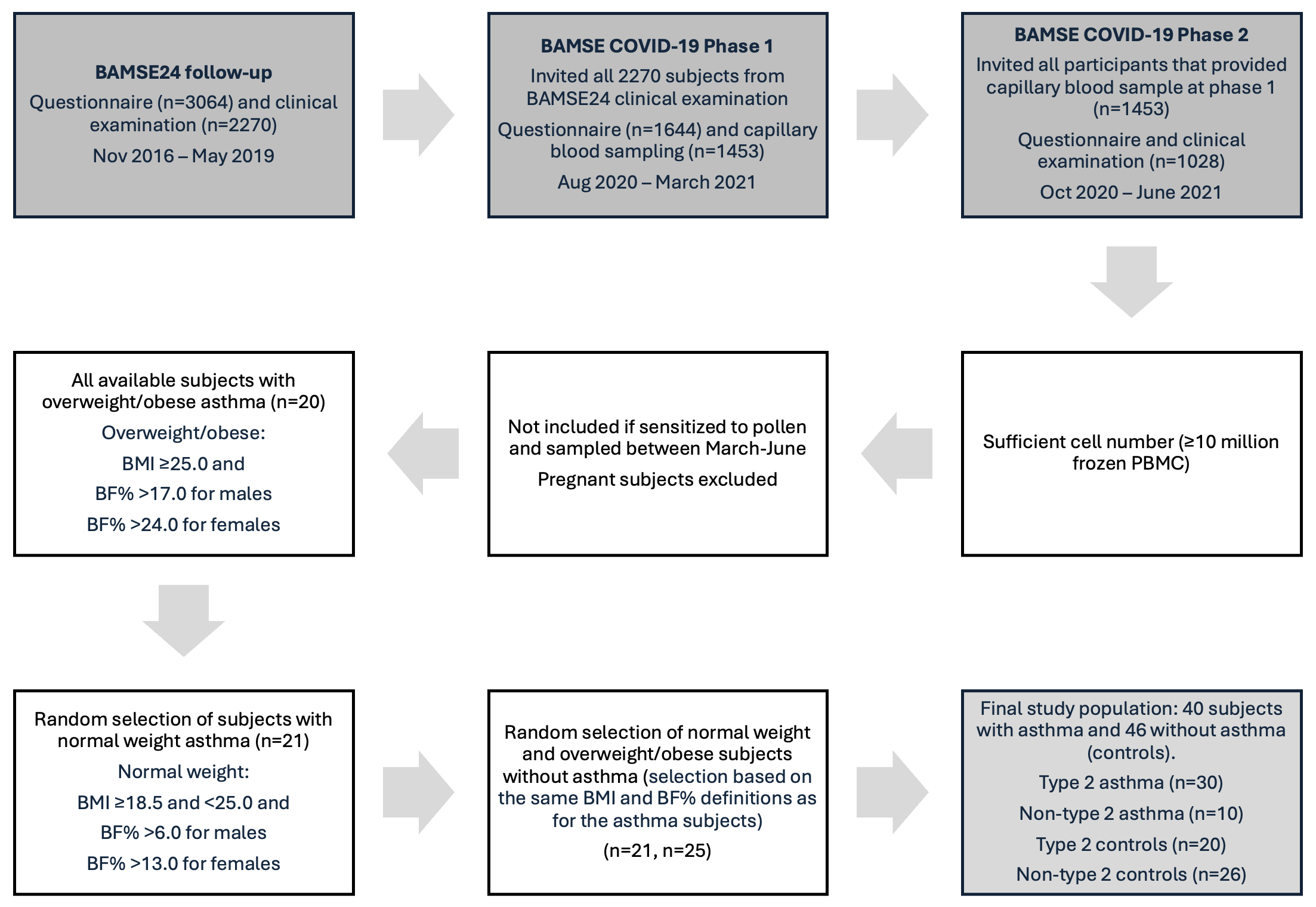
**

**Supplementary Figure S2.** Dendrograms of ILC/NK, CD4, and CD8 parameters after hierarchical clustering. The most relevant subpopulations within each cell type were selected by hierarchical clustering, employing the Ward D2 method on an Euclidean distance matrix for dimensionality reduction. The resulting dendrograms were then cut at a height of 20 which was determined by visual inspection. Hierarchical clustering produced 16 ILC/NK cell clusters, 16 CD4 T cell clusters, and 12 CD8 T cell clusters. ILC= innate lymphoid cell.

**
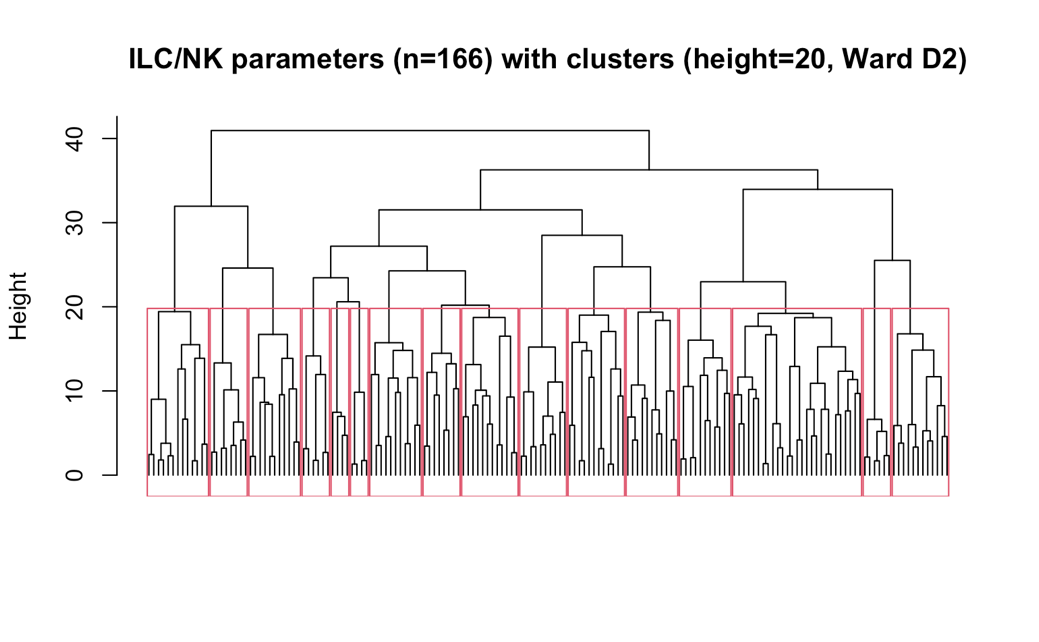

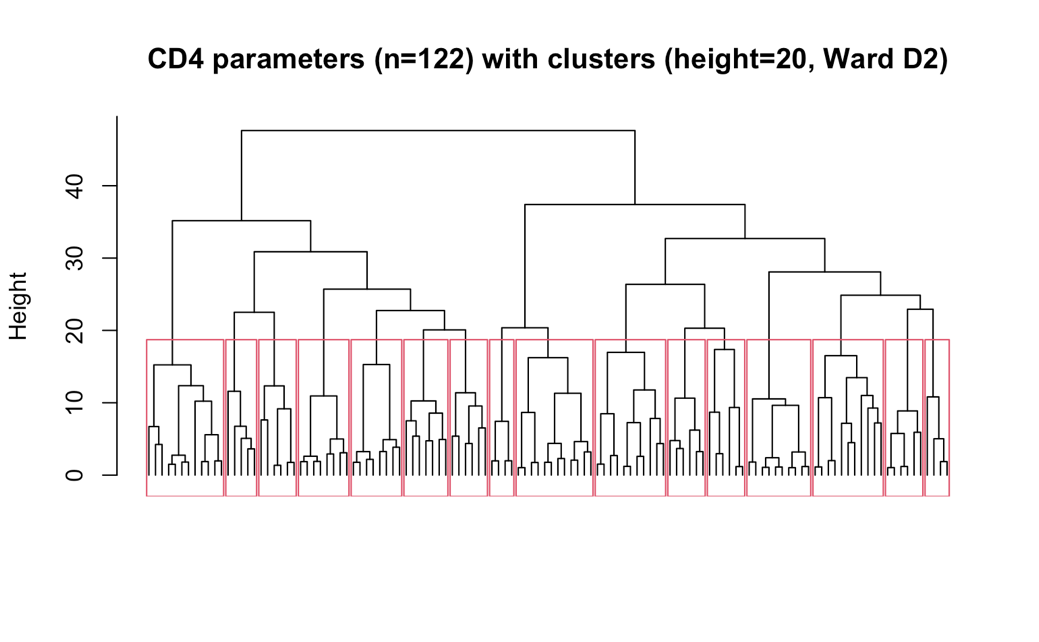

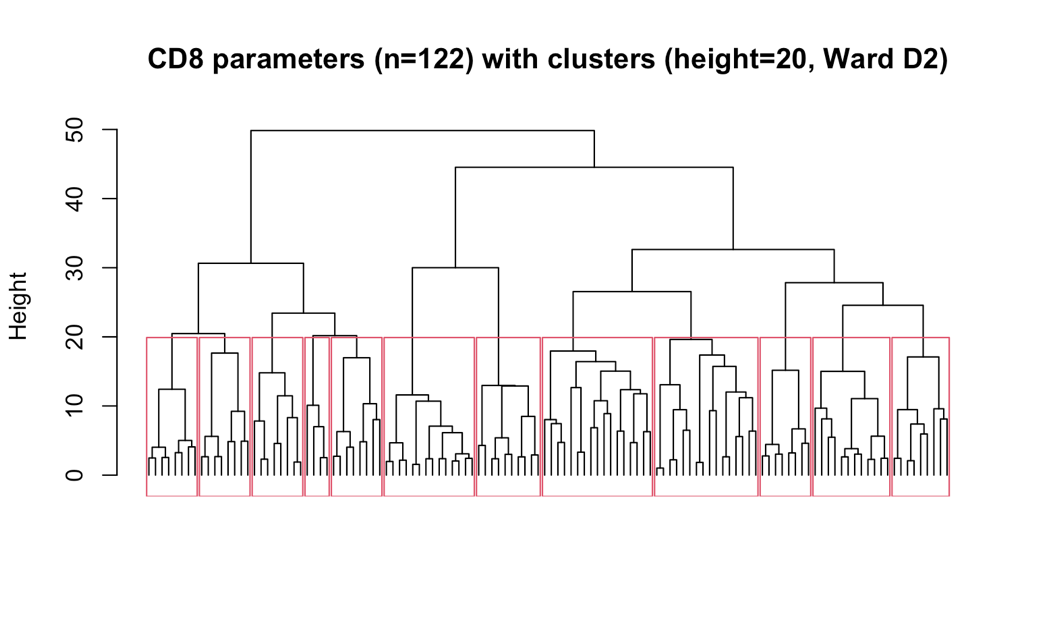
**

**Supplementary Figure S3.** Significant individual subpopulations in CD4 cluster 16 significantly differentiate between type 2 and non-type 2 asthma (comparison set 1) and between type 2 and non-type 2 controls (comparison set 2). P-value determined by Mann-Whitney U test. Ns: non-significant p-value (*p* > 0.05); *: p ≤ 0.05, **: p ≤0.01. TCM: T central memory cell, TEM: T effector memory cell.

**
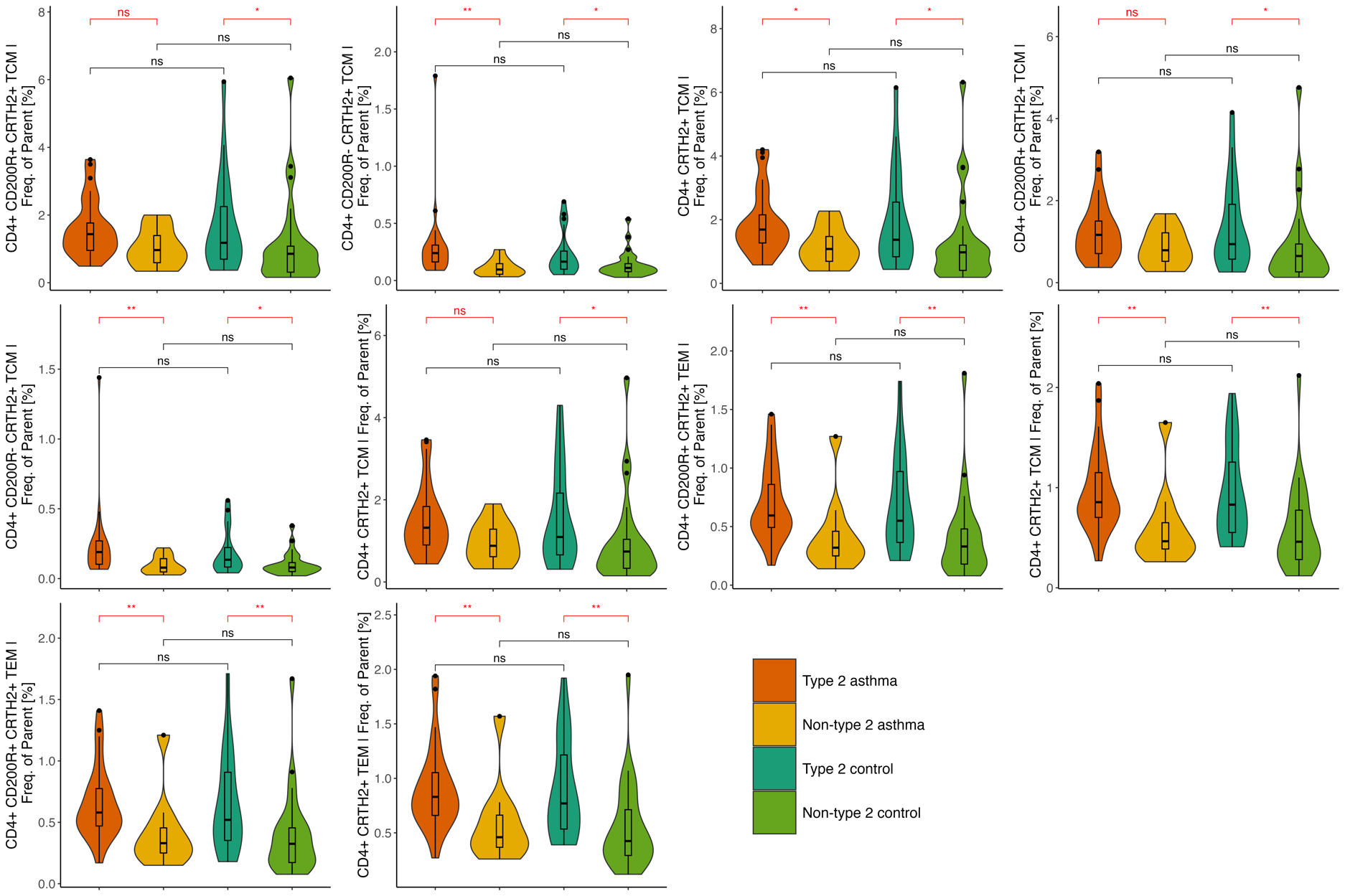
**

**Supplementary Figure S4.** Significant individual subpopulations in ILC/NK cluster 5 significantly differentiate between type 2 and non-type 2 asthma (comparison set 1). P-value determined by Mann-Whitney U test. Ns: non-significant p-value (*p* > 0.05); *: p ≤ 0.05, **: p ≤0.01. ILC2: Type 2 innate lymphoid cell, ILCp: innate lymphoid cell precursor.

**
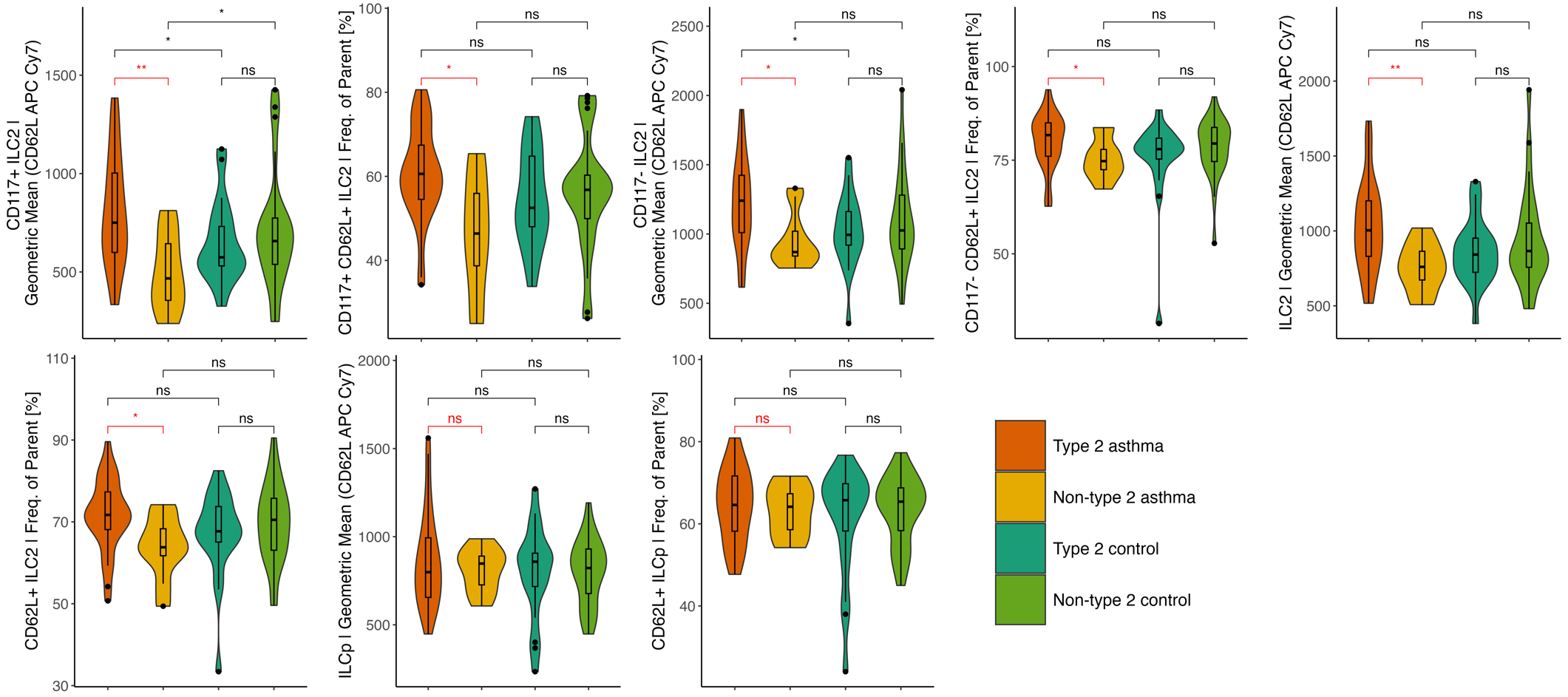
**

**Supplementary Figure S5.** Significant individual subpopulations in CD4 cluster 9 significantly differentiate between type 2 and non-type 2 asthma (comparison set 1). P-value determined by Mann-Whitney U test. Ns: non-significant p value (*p* > 0.05); *: p ≤ 0.05, **: p ≤0.01, ***:p ≤0.001. TCM: T central memory cell, TEM: T effector memory cell, TEMRA: T effector memory RA-expressing cell.


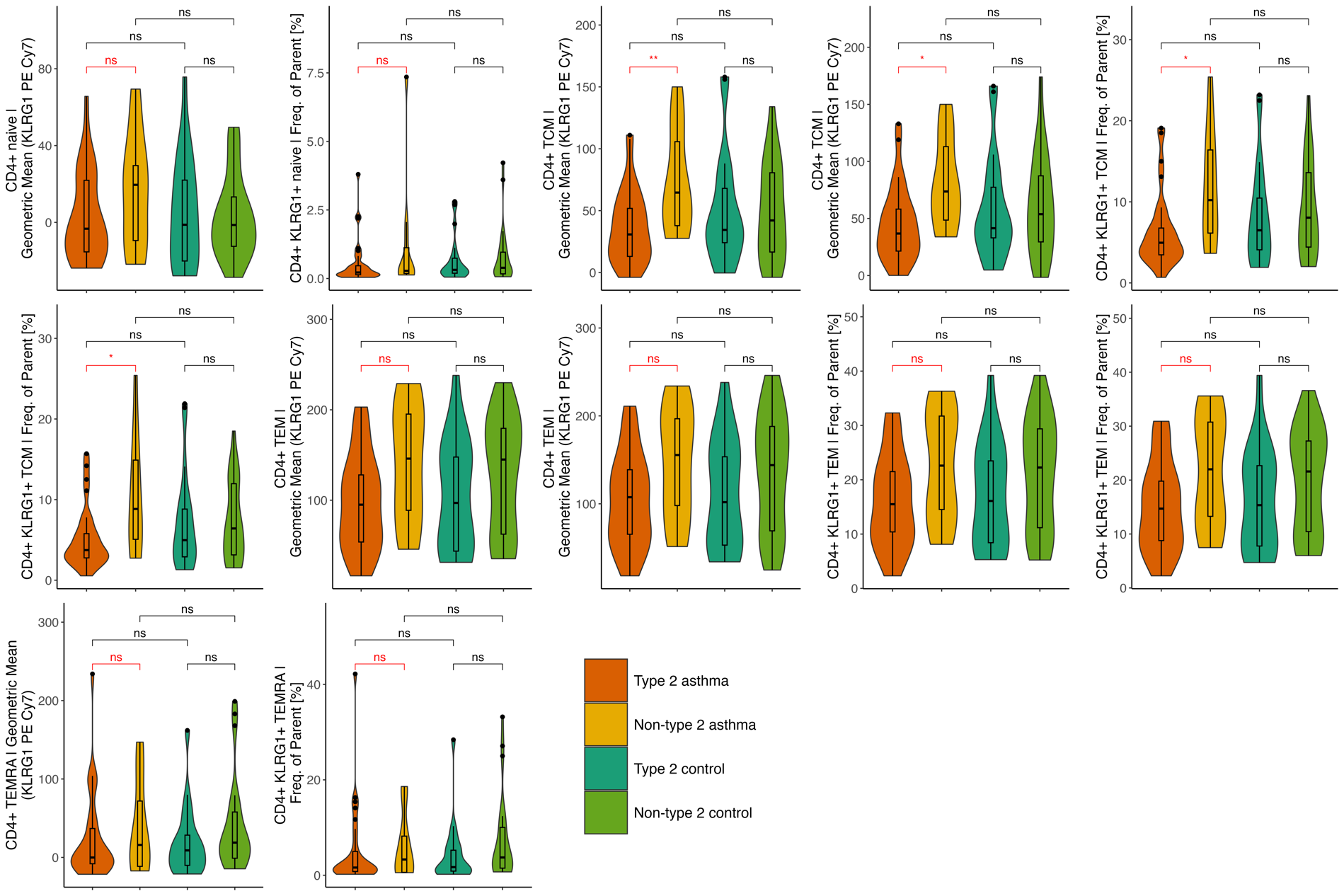


**Supplementary Figure S6.** Remaining significant associations from logistic regression models presented in Table 2 (and not presented in Figures 1 and 2). Note that the y-axis is inverse normally transformed. A. ILC/NK cluster 3 representative variable (RV) is associated with type 2 controls. B. CD8 cluster 6 RV is associated with type 2 asthma (p value 0.043). C: CD4 cluster 14 RV is associated with non-type 2 asthma (p value 0.046).

**
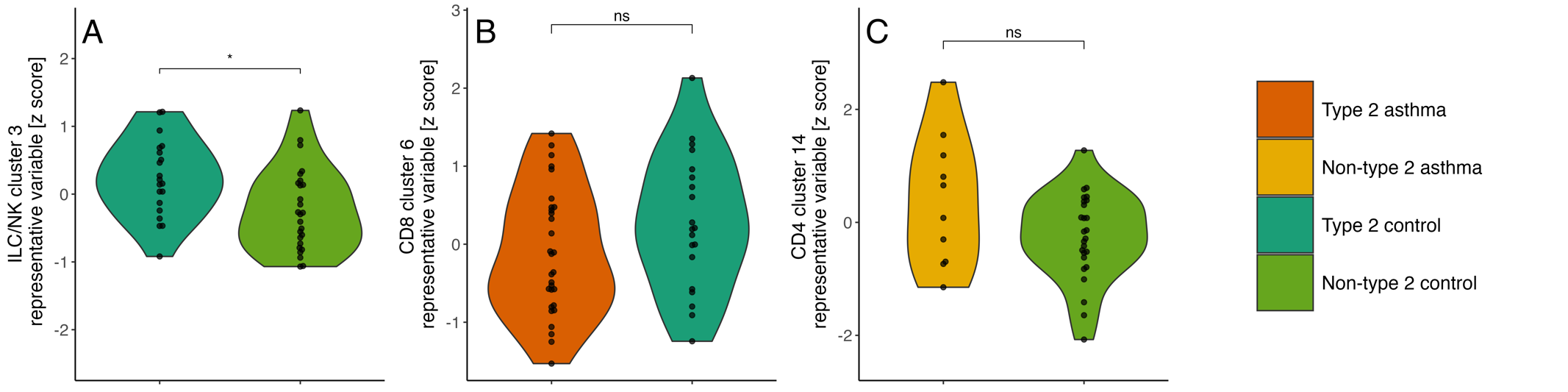
**

**Supplementary Figure S7.** Significant individual subpopulations in ILC/NK cluster 3 that differentiate between type 2 and non-type 2 controls (comparison set 2). P-value determined by Mann-Whitney U test. Ns: non-significant p-value (*p* > 0.05); *: p ≤ 0.05. ILC(2): (type 2) innate lymphoid cell, ILCp: innate lymphoid cell precursor.





**Supplementary Figure S8.** CD8 cluster 6 RV is associated with T2 asthma (comparison set 3), but no significant differences in individual subpopulations were observed between T2 asthma and T2 controls. P-value determined by Mann-Whitney U test. Ns: non-significant p-value (*p* > 0.05); *:p ≤ 0.05. TCM: T central memory cell, TEM: T effector memory cell, TEMRA: T effector memory RA-expressing cell.

**
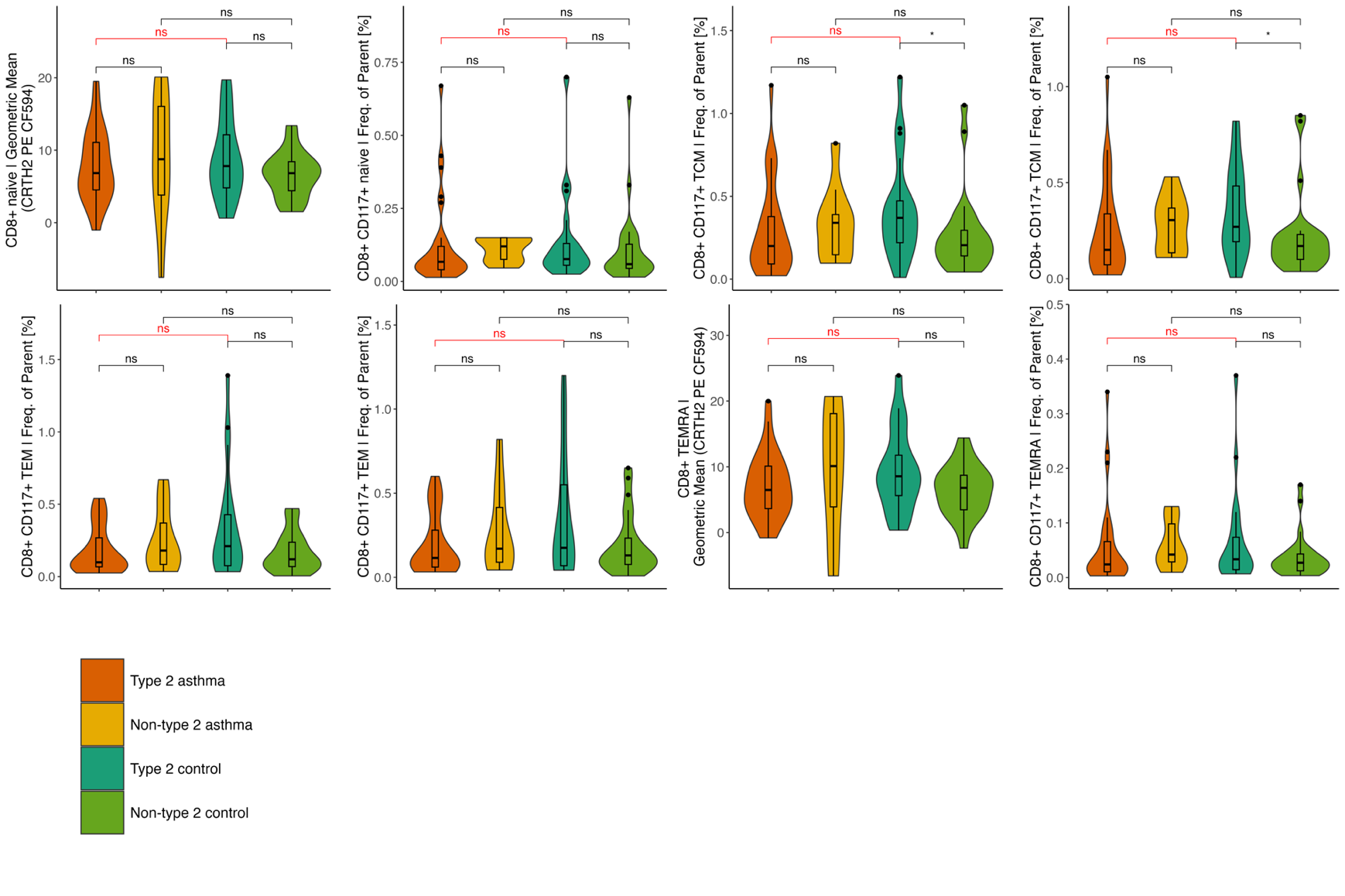
**

**Supplementary Figure S9.** Significant individual subpopulations in CD8 cluster 2 significantly differentiate between non-type 2 asthma and non-type 2 controls (comparison set 4). P-value determined by Mann-Whitney U test. Ns: non-significant p-value (*p* > 0.05); *: p ≤ 0.05, **: p ≤0.01.

**
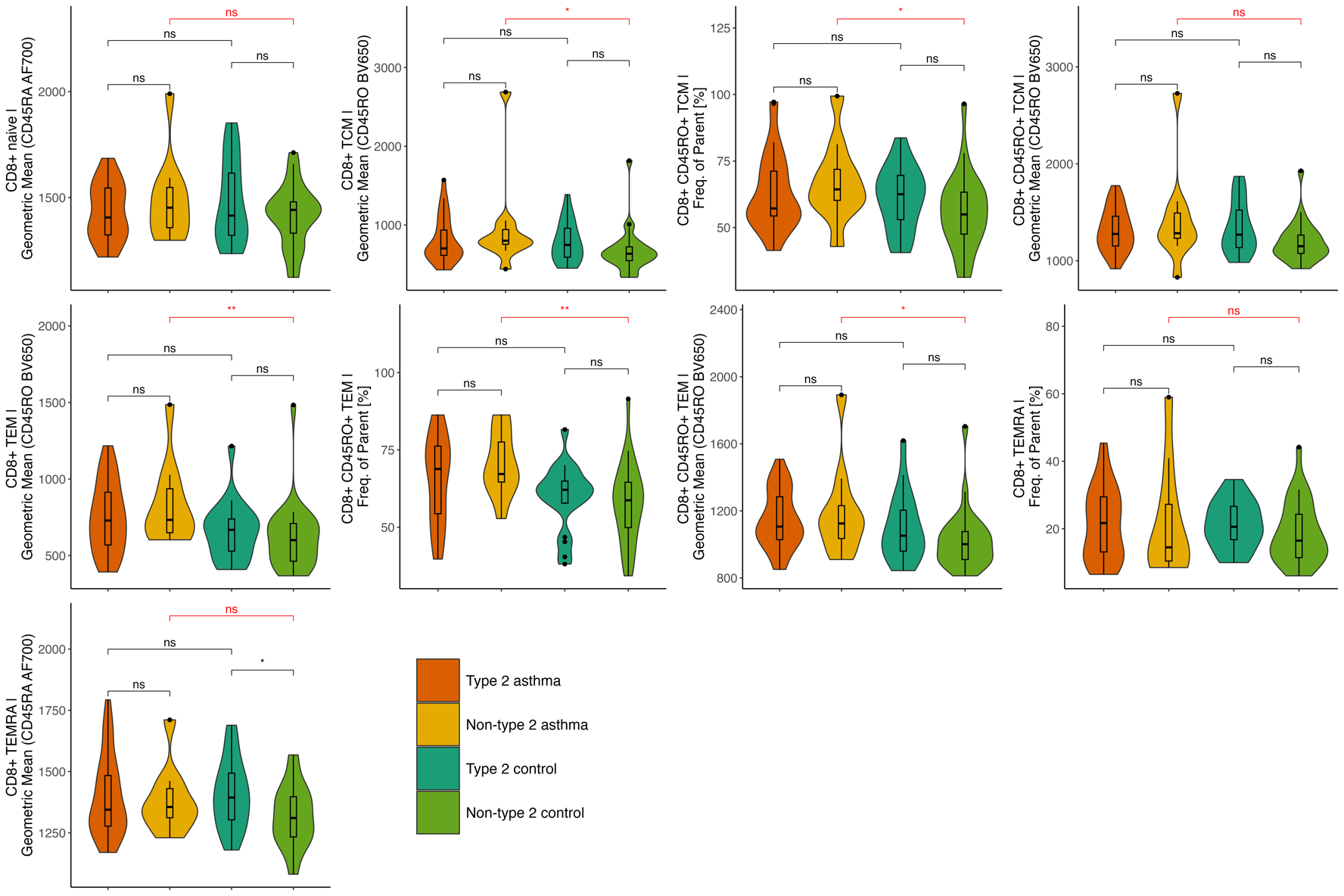
**

**Supplementary Figure S10.** Significant individual subpopulations in ILC/NK cluster 7 significantly differentiate between non-type 2 asthma and non-type 2 controls (comparison set 4). P-value determined by Mann-Whitney U test. Ns: non-significant p-value (*p* > 0.05); *: p ≤ 0.05. ILC2: type 2 innate lymphoid cell.

**
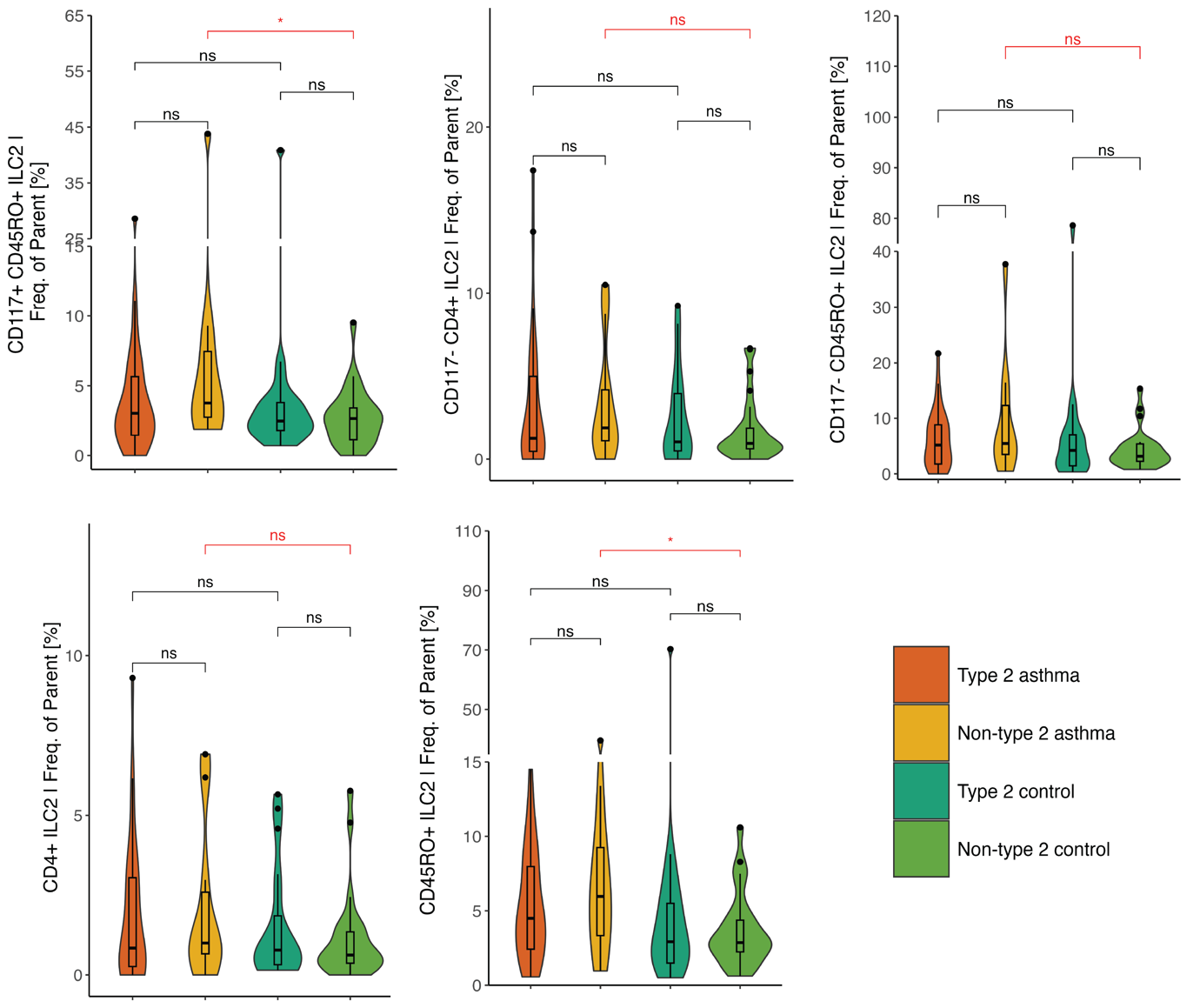
**

**Supplementary Figure S11.** CD4 cluster 14 RV is associated with non-type 2 (non-T2) asthma (comparison set 4) but no significant differences in individual subpopulations were observed between non-T2 asthma and non-T2 controls. P-value determined by Mann-Whitney U test. Ns: non-significant p-value (*p* > 0.05). TCM: T central memory cell, TEM: T effector memory cell.

**
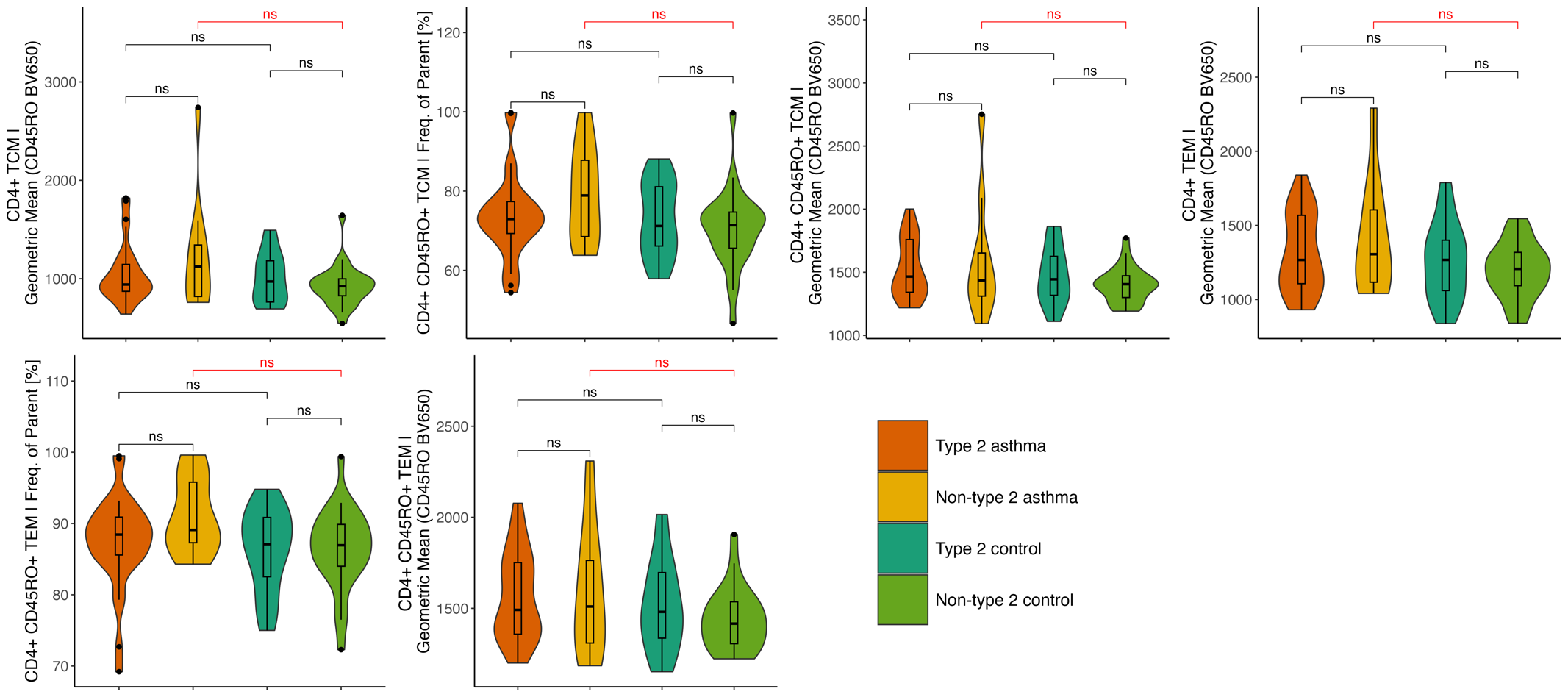
**

**Supplementary Figure S12.** Type 2 endotypes over a period of 1-5 years, measured twice in the same 86 individuals, and comparison between type 2 endotypes at the BAMSE COVID-19 follow-up with and without the addition of Fractional Exhaled Nitric Oxide (FeNO) to the definitions (FeNO cut-off 25 parts per billion). NA=missing data.

**
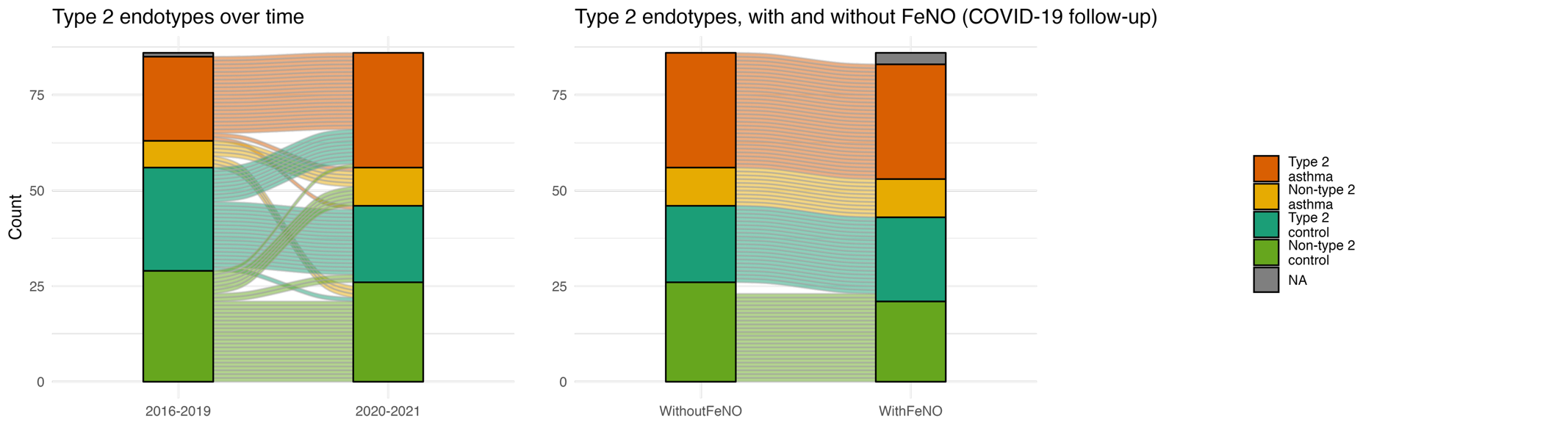
**
